# Medicinal Plants Used for Treating Mild Covid-19 Symptoms Among Thai Karen and Hmong

**DOI:** 10.3389/fphar.2021.699897

**Published:** 2021-07-20

**Authors:** Methee Phumthum, Varangrat Nguanchoo, Henrik Balslev

**Affiliations:** ^1^ Department of Pharmaceutical Botany, Faculty of Pharmacy, Mahidol University, Bangkok, Thailand; ^2^ Sireeruckhachati Nature Learning Park, Mahidol University, Nakhon Pathom, Thailand; ^3^ Department of Biology, Faculty of Natural Science, Aarhus University, Aarhus, Denmark

**Keywords:** cough, diarrhea, fever, ethnobotany, headache, traditional knowledge

## Abstract

**Background:** The COVID-19 pandemic is causing many severe problems globally, and it is not known for how long it will last. The only hope we have for dealing with the problem is to produce sufficient vaccines and administer them efficiently. However, the current demand for vaccines greatly exceeds the supply, and many people will suffer from the disease for still some time. Moreover, the period for immunity obtained by the vaccines remains unknown, and we cannot predict how long the world will suffer the COVID-19 infections. Therefore, there will be a continued demand for treatments of its symptoms. An alternative solution for providing such treatment is the use of traditional medicinal plants.

**Aims:** To document medicinal plants used by Hmong and Karen in Thailand to treat mild symptoms of COVID-19.

**Methods:** Traditional knowledge about ethnomedicinal plants used by Hmong and Karen in Thailand for treating mild symptoms listed by WHO as associated with COVID 19, was collected in field interviews and extracted from the literature.

**Results:** We identified 491 plant species used medicinally by both ethnic groups to treat fever, cough, diarrhea, muscle pain and ache, rash, headache, sore throat, and conjunctivitis. Of the 491 species 60 were mentioned at least five times in the literature or in our field data. Of these 60 species, we propose the most commonly used ones for treatments of mild COVID-19 symptoms. Ten of these most commonly mentioned species were used for treatments of fever, nine for treatment of cough, four for treatment of diarrhea, two for treatment of rash, and a single species was used to treat muscle pain and headache.

**Conclusion:** This study suggests alternative treatments for mild symptoms of COVID-19 with medicinal plants that are traditionally used by the ethnic minority groups of the Hmong and Karen in Thailand. Although COVID-19 is a new disease, its mild symptoms are shared with many other diseases. Traditional knowledge on medicinal plants used by the Thai Karen and Hmong could help in the treatments of these symptoms associated with COVID-19. Many of the proposed plants were used abundantly by both ethnic groups, and other studies on biological activities support their efficacy in such treatments.

## Introduction

In December, 2019, the emergence of a novel human virus caused respiratory system disorders in a population in Wuhan, China. Subsequently, the infection spread globally (Li et al., 2020; Zhu et al., 2020) and the virus was named SAR-CoV2 and the disease became known as COVID-19. The virus created havoc globally and it affected many people in all countries of the world. In mid-April, 2021, over three million people had died out of the 140 million who had contracted the disease worldwide and still, over 800,000 new cases are being reported daily (WHO, 2021b). The symptoms of COVID-19 vary. The most common symptoms are fever, dry cough, and fatigue. Less common symptoms are aches and pains, sore throat, diarrhea, conjunctivitis, headache, loss of taste or smell, skin rash, and discoloration of fingers and toes. The most serious symptoms are difficulty of breathing and shortness of breath, chest pain, and pressure, and loss of speech or movement (WHO, 2021a). The fear of this disease has led to the development of several vaccines, but many people are afraid of side effects from being vaccinated. It is worrying, that the current production rate of COVID-19 vaccines cannot meet the global demand. We are still learning about the COVID-19 vaccines and their efficacy in preventing new variants of the virus. Preliminary data show that some vaccines work against some variants but that they are less effective in preventing others (CDC, 2021). Another concern is that the duration of protection after getting vaccinated is not well documented. It is possible that vaccines could only protect for a short period and that vaccines would have to be given regularly, in the same way as vaccines that protect from other fevers and flues, hepatitis A and B, Tdap, and others. The worst-case scenario, even with effective vaccines that can prevent fatalities, is that the mild COVID-19 symptoms will remain a threat to our race for an unknown period of time.

In Thailand, even though there are few cases of COVID-19 infections and deaths compared to many other countries, the pandemic strongly affects cultures, economy, and happiness of the population. Thailand has only a few million doses of vaccines which is very low compared to the needs of approximately 70 million people. A similar excess in demand is found all over the world. According to UNICEF, 20% of the world population will be vaccinated by 2021 and vaccination of the entire world population might not be achieved until 2023 or later. Additional demands for vaccines depend on how long immunity will last (UNICEF, 2020). This leads to uncertainty about the time it will take to fight this disease, and especially to combat the mild symptoms of infections. Many people who have not been vaccinated are at risk of the infections and most infected people would suffer from the COVID-19 showing only mild symptoms. Therefore, we need medicines to treat such mild symptoms. Locally used medicinal plants could be one of the choices for patients infected, especially in developing countries that already extensively use medicinal plants in their primary health care (WHO, 2002). The demand for medicinal plants has increased during the COVID-19, and the used plants were mostly associated with COVID-19 treatments (Khadka et al., 2021). However, because COVID-19 is a novel disease, there are no specific medicinal plants used to treat its symptoms. Uses of medicinal plants for the treatments is mostly based on medicinal plants used to treat symptoms related to the COVID-19 symptoms. A study, that evaluated risk/benefits of medicinal plants used as adjuvant of COVID-19 treatments, found that many herbal species are safe to use (Silveira et al., 2020).

Ethnobotany is a young discipline in Thailand, but even so, the traditional medicinal plant knowledge of many of the ethnic minority groups has been studied in some detail (Phumthum et al., 2018; Phumthum, 2020). The Karen and the Hmong are the largest ethnic minorities in Thailand (HHDC, 2018). Both groups have vast traditional knowledge of medicinal plant uses (Phumthum, 2020; Phumthum et al., 2020). Traditional knowledge of medicinal plants used by these ethnic minorities is mostly unique to each group (Phumthum and Balslev, 2019). Uses of the same medicinal plants for the same treatments by people from different ethnic groups suggest efficacy of the plants (Saslis-Lagoudakis et al., 2011). Thus, cross-cultural comparison of the traditional knowledge of medicinal plants by the Hmong and the Karen in Thailand is useful for identifying medicinal plants that could be effective for treatments of mild COVID-19 symptoms. This leads to the aim of this study, which is to identify medicinal plants that can be proposed for treatments of mild COVID-19 symptoms. The proposal is based on traditional knowledge of Thai Hmong and Karen about the medicinal plants they use for treatments of symptoms similar to mild COVID-19 symptoms.

## Methods

Ethnomedicinal data from 31 Karen villages and 19 Hmong villages were derived from already published data and new data from six Hmong villages were collected in field studies by the authors.

### Published Data

**FIGURE 1:**
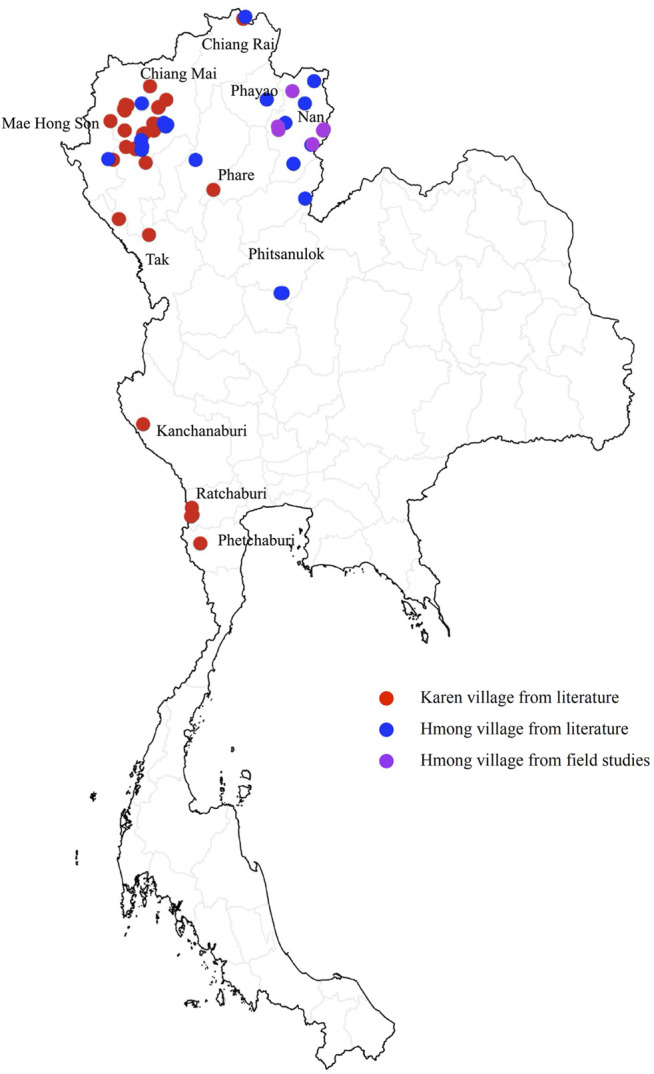
Locations of 56 Thai Hmong and Karen villages providing ethnomedicinal plant knowledge for treatments of symptoms related to mild Covid-19 symptoms in this study.

Available published data on ethnomedicinal plants used for treatments of mild COVID-19 related symptoms, such as fever, dry cough, fatigue, aches and pains, sore throat, diarrhea, conjunctivitis, headache, loss of taste or smell, skin rash, discoloration of fingers or toes (WHO, 2021a) were extracted from already published ethnobotanical studies. The inclusion criteria were: 1) The study must use accepted standard ethnobotanical methods (Martin, 1995) for collecting data; 2) all used species must be identified by their scientific names using standard botanical nomenclature; 3) the studies must have been conducted in Hmong and Karen villages in Thailand; 4) at least one species in the list must have been used for treatment of one of the mentioned mild COVID-19 symptoms; 5) the study must have been published between 1990 and 2020; 6) repeated data, such as data in a student thesis that was subsequently published in journal articles were combined. Using these criteria 33 publications were available as data sources and they originated from 19 Hmong villages and 31 Karen villages (Figure 1, Supplementary Data).

### Field Study

To compensate for the smaller number of Hmong villages in the literature-based data, we conducted additional field studies to add information about traditional ethnomedicinal knowledge from six Hmong villages in Nan Province Thailand (Figure 1). Ethical approval was obtained from Chiang Mai University Research Ethics Committee with the certificate of approval number COA No. 020/61 (extended to April 19th, 2021). In the same way as the literature-based data, the data collected in the field covered plants used for treatments of the mentioned mild COVID-19 symptoms. Sixteen key respondents, who were local healers that used medicinal plants, were selected for semi-structured interviews using purposive and snow ball sampling. The studies were conducted from March 2020 to February 2021. The respondents were asked to show the plants that they used as medicine to treat the mentioned symptoms. The used plants were photographed and herbarium samples were collected for identification and documentation. The plants were identified by the authors using keys and plant descriptions in the Flora of Thailand and neighboring areas. Plant voucher specimens were deposited at the herbarium of the Department of Pharmaceutical Botany Mahidol (PBM) and Queen Sirikit Botanic Garden Herbarium (QBG).

Finally, scientific plant names and families from both published data and our field study were standardized to follow the World Checklist of Vascular Plants (WCVP) website (Kew, 2020).

### Important Species

Important medicinal species for treatments of mild COVID-19 symptoms were evaluated by using species Use Value (UV_s_) (Phillips and Gentry, 1993). The Use Value is calculated using the following formula:

$$UVs=\;\frac{\Sigma Uis}{n}$$

where *Uis* is the number of use reports mentioned by each respondent (informant) *i* for a given species while *n* is the total number of interviewed respondents. In this study, we included both data from previous studies and the data from our recent field observation. It was difficult to identify that which respondent cited which use. We standardized the term “respondent”, which refer to a person citing a used species to “pseudorespondent” which refer to an area providing information of a used species (Phumthum et al., 2018). Therefore, the UV mentioned in this study is calculated form pseudorespondents. High UV indicates that the plants were frequently used by the pseudorespondent and implies that the plant would have potential for treatments.

## Results

### Numbers of Use Reports

**FIGURE 2:**
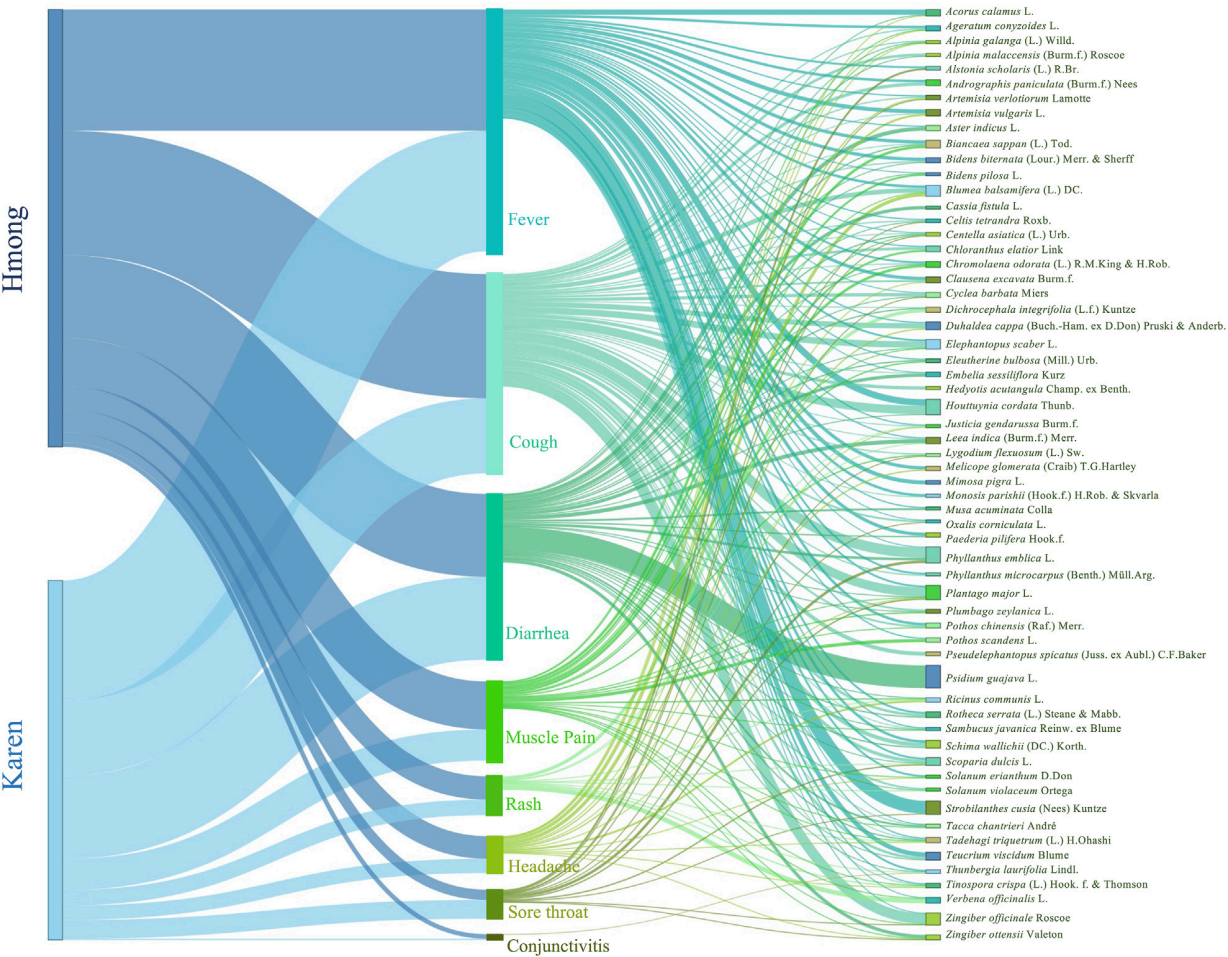
The links of use reports of Hmong and Karen in Thailand for the 60 medicinal plants most used to treat mild symptoms related to COVID-19.

Our data include information from 56 villages (Hmong 25, Karen 31) (Figure 1) and 1230 use reports (Hmong 675, Karen 555) (Figure 2, Supplementary Table 1, supplementary materials) and 491 species (Hmong 269, Karen 297) that the people from these ethnic groups used for treatments of mild COVID-19 symptoms. Not all the above mentioned mild COVID-19 symptoms were covered in our data. We only found plants used for treatments of fever, cough, diarrhea, muscle pain, rash, headache, sore throat, and conjunctivitis, whereas the other mild COVID-19 symptoms were not treated with any plants. The number of use reports for different treatments were similar in the Hmong and Karen data. Fever was the most commonly treated symptom followed by cough, diarrhea, muscle pain, skin rash, headache, and sore throat, respectively. Treatment of conjunctivitis had the lowest numbers of use reports among both Hmong and Karen (Figure 2).

### Most Used Species

Although the published and additional field studies of both ethnic groups together listed 491 species used for treatments of mild symptoms related to the COVID-19, sixty species had high use values (UVs) (Supplementary Table 1, supplementary materials). These 60 species accounted for almost half of the total number of use reports (Figure 2). *Psidium guajava* L. had the highest number of use reports (35) followed by *Phyllanthus emblica* L. (25), *Houttuynia cordata* Thunb. (24), *Plantago major* L. (22), and *Strobilanthes cusia* (Nees) Kuntze (21). Eleven species (*Zingiber officinale* Roscoe, *Blumea balsamifera* (L.) DC., *Elephantopus scaber* L., *Biancaea sappan* (L.) Tod., *Duhaldea cappa* (Buch.-Ham. ex D.Don) Pruski and Anderb., *Schima wallichii* (DC.) Korth., *Scoparia dulcis* L., *Teucrium viscidum* Blume, *Acorus calamus* L., *Artemisia vulgaris* L., and *Leea indica* (Burm.f.) Merr.) had 10–20 use reports (Figure 2).

**FIGURE 3:**
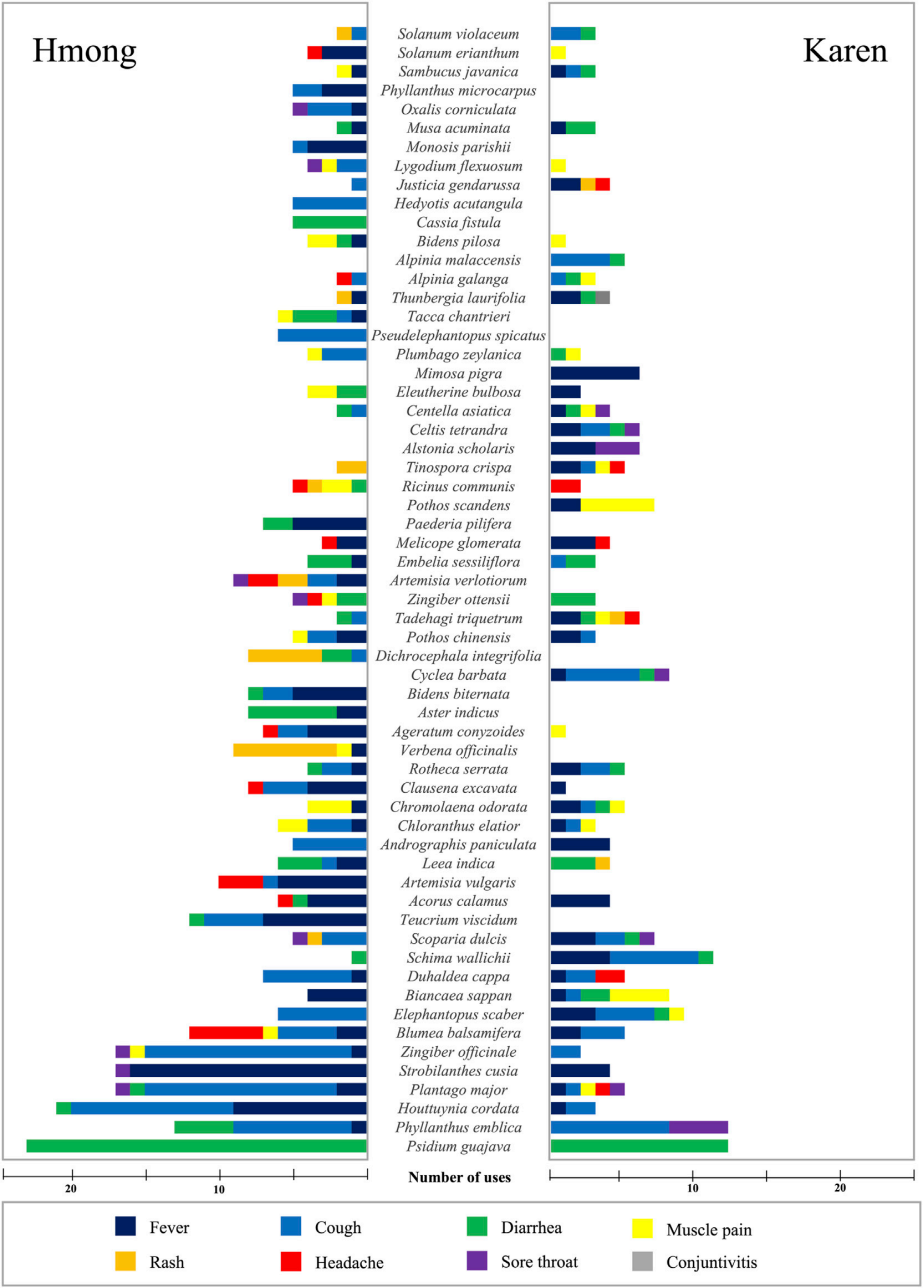
The numbers of use reports for the 60 species which had high UVs of the Hmong and the Karen in Thailand for treatments of eight mild COVID-19 symptoms. Bars in “Hmong” areas represent the number of each species’ use reports by the Hmong while the bars in the “Karen” area represent the species use reports by the Karen.

Hmong and Karen people had different ethnomedicinal knowledge about the 60 most used species. The Hmong reported 54 species of which 15 were not mentioned by the Karen. And the Karen reported 45 of the 60 most used species and six of them were not used by the Hmong. However, there were many species used by both the Hmong and the Karen that had abundant use reports for treatments of the same symptoms. *Psidium guajava* had one hundred percent of use reports for treatments of diarrhea by both Hmong and Karen people. Both ethnic groups also agreed that *Phyllanthus emblica* was useful for treating cough and fever. Approximately 94 percent of the use reports of *Strobilanthes cusia* from the Hmong and 100 percent from the Karen mentioned the use for treatment of fever. High percentages of uses from both ethnic groups presented many species for the same treatments, namely *Zingiber officinale* (cough), *Blumea balsamifera* (cough), *Elephantopus scaber* (cough), *Acorus calamus* (fever), *Zingiber ottensii* (diarrhea), and *Melicope glomerata* (fever) (Figures 2, 3).

Many species were used for treatments of specific symptoms. The Hmong used *Teucrium viscidum* to treat fever and cough, *Artemisia vulgaris* to treat fever and headache, *Verbena officinalis* and *Dichrocephala integrifolia* to treat rashes, *Aster indicus* and *Cassia fistula* to treat diarrhea, *Bidens biternata, Monosis parishii* and *Paederia pilifera* to treat fever, *Pseudelephantopus spicatus* and *Hedyotis acutangula* to treat cough. The species that were commonly used only by the Karen for treatments of specific symptoms were *Cyclea barbata* and *Alpinia malaccensis* that were used to treat cough, *Pothos scandens* used to treat muscle pain and *Mimosa pigra* which was uused to treat fever (Figure 3).

### Species Used for Multiple Treatments

Many species listed in this study were used for treatments of several symptoms, sometimes as many as five (Figure 2). *Plantago major* was used by the Hmong to treat four symptoms and by the Karen to treat five different symptoms. Other species used for treatments of five symptoms included *Artemisia verlotiorum* (Hmong), and *Tadehagi triquetrum* (Karen). Many species were used to treat four symptoms by Hmong (*Zingiber officinale, Blumea balsamifera, Zingiber ottensii, Ricinus communis, Tacca chantrieri*) and Karen (*Elephantopus scaber, Biancaea sappan, Scoparia dulcis, Cyclea barbata, Tinospora crispa, Celtis tetrandra,* and *Centella asiatica*) (Figure 3).

## Discussion

Because COVID-19 is a new emerging disease, there are no records of medicinal plants specifically used to treat the disease. This study presents medicinal plants for which traditional knowledge in 31 Karen and 25 Hmong villages in Thailand show that they are used to treat disease with symptoms that are similar to mild COVID-19 symptoms. Although there are many mild symptoms of the COVID-19 (WHO, 2021a), we found traditional knowledge only for treatments of some of them (fever, cough, diarrhea, muscle pain, rash, headache, sore throat, and conjunctivitis). These are common symptoms that people experience every year, especially seasonal symptoms such as fever, cough, headache, and sore throat. A previous study showed that Thai Karen had tremendous knowledge of medicinal uses for treatments of fever (Phumthum and Sadgrove, 2020). Diarrhea is usually found among Thai rural population because of the consumption of unsanitary or very sour and spicy food, and body aches and pains are commonly found among laborers. These are all common ailments that Thai people use medicinal plants to treat (Phumthum et al., 2018).

Our comparison of UV values obtained in different localities should be interpreted with some caution since the same UV values may be obtained from different use numbers. For instance, in four villages the proportions of plants used for treatment of fever were 2/10, 40/200, 6/30, and 5/25 giving the same UV value of 0.2 but differing in variance of the data. We also used the concept of pseudoinformant (respondent) to represent the averages obtained in studied villages because data on individual respondents were not available. So, in that case the UV value represent the proportion of villages studied rather than the proportion of respondents who gave a particular answer, which obviously also must be interpreted with caution. Nevertheless, the UV values obtained using the pseudoinformant concept is a reflection of how widespread a use is and therefore may be related to the efficacy of the treatments (Saslis-Lagoudakis, 2011). Regardless of these caveats, our study showed 60 of 491 species had high UVs. It is likely that the majority of these 60 commonly used species are biomedically effective because almost all of them belong to important medicinal plant families in Thailand and elsewhere (Moerman, 1996; Leonti et al., 2003; Bourbonnais-Spear et al., 2005; Bennett and Husby, 2008; Saslis-Lagoudakis et al., 2011; Weckerle et al., 2011; Ford and Gaoue, 2017; Kew, 2017; Phumthum et al., 2019). Cross-cultural uses of medicinal plants of a single species for the same purpose suggests that the plant could be a good candidate for bioprospecting studies (Saslis-Lagoudakis et al., 2011). This study shows that the majority of the 60 commonly used species were used for different purposes by Hmong and Karen in Thailand (Figure 3) and a previous study showed the traditional knowledge is often unique to these ethnic groups (Phumthum and Balslev, 2019). Thus, the same uses of the same plants by the Hmong and the Karen suggests that they are effective plants for treatments. There are many mild COVID-19 symptoms, but the most common ones are fever, dry cough, and fatigue (WHO, 2021a). Both the Hmong and the Karen used *Strobilanthes cusia* and *Acorus calamus* extensively for treating fever, *Phyllanthus emblica* for treating cough and fevers, *Zingiber officinale*, *Blumea balsamifera*, and *Elephantopus scaber* for treating cough (Figure 3). Other less common symptoms of the COVID-19 are aches and pains, sore throat, diarrhea, and conjunctivitis (WHO, 2021a). The species most used to treat these symptoms was *Psidium guajava,* which was used in similar ways and for the same purpose by both ethnic groups. *Zingiber ottensii* was also used by both the Hmong and the Karen to treat diarrhea.

Because the Hmong and the Karen have different histories and cultures, some species were used by both groups but in totally different ways. The Hmong used *Andrographis paniculata* only to treat cough while the Karen used the same species only to treat fever (Figure 3). Interestingly, those species had high numbers of use reports for treatments of various symptoms. All use reports of *Mimosa pigra* were for treatment of fever by the Karen whereas the Hmong used *Cassia fistula* and *Hedyotis acutangula* for treatments of diarrhea and cough, respectively, (Figure 3). This suggests that these unique intensively used species could be effective medicinal plants for treatments of the mild COVID-19 symptoms.

We prioritized the species for potential further use by the following criteria: 1) plants commonly used by both ethnic groups for treatment of the most common mild COVID-19 symptoms; 2) plants commonly used by either ethnic groups for common symptoms; 3) plants commonly used by both ethnic groups for less common symptoms; and 4) plants commonly used for treatments of less common symptoms used by either ethnic group. Using these criteria, we could rank the top proposed species. *Strobilanthes cusia* and *Acorus calamus* ranked as the top species for treatments of fever. Other studies have shown that a mixture of medicinal plants with *S. cusia* extract inhibited influenza virus growth in a mice model (Cervi et al., 2006). Another study showed that tryptanthrin and indigodole B from *S. cusia* had bioactivity against human coronavirus (HCoV-NL63) (Tsai et al., 2020). *Acorus calamus* is also used to treat fever by other ethnic groups in Thailand and elsewhere (Sharma et al., 2014; Panyadee et al., 2019; Kyaw et al., 2021). *Phyllanthus emblica* has been used to treat cough and several symptoms in Ayurvedic, Turkish, Unani, and Tibetan medicines for centuries. The plant produces various kinds of secondary metabolites and the compounds act in various therapeutic treatments (Ahmad et al., 2021). Many anti-cough lozenge products from this plant are sold in Thailand. However, there is only limited scientific evidences that the plant is effective for treatment of fever. *Zingiber officinale* was evaluated as a medium benefit, medium overall safety, and promising benefit/risk herbal medicine as adjuvant COVID-19 symptomatic therapy (Silveira et al., 2020). An experiment in chick embryos showed that the plant had anti-avian influenza activity (Ahmed et al., 2017). *Blumea balsamifera* and *Elephantopus scaber* listed as Thai ethnomedicinal plants with high use values for treatment of various infections (Phumthum et al., 2018). The plants produce a numbers secondary metabolites (Fu et al., 2020; Hanh et al., 2021), and they have many biological activities (Wang and Yu, 2018; Yufri et al., 2019), but there is only limited scientific evidences for pharmacological activities of these plants for treatment of mild COVID-19 symptoms. *Psidium guajava* was the most interesting plant for treatment of diarrhea which is a less common mild COVID-19 symptom. There is abundant scientific evidences that the plant produces large amounts of tannins (Anbuselvi and Rebecca, 2017), and that it is effective in the treatment of diarrhea (Hirudkar et al., 2020; Lu et al., 2020). *Zingiber ottensii,* a candidate for treatments of diarrhea, is much used in Thailand as a medicine for treatments of symptoms related to digestive system disorders (Phumthum and Balslev, 2020).

The medicinal plants used only by the Hmong or only by the Karen are also interesting candidates as medicinal plants for treating the mild COVID-19 symptoms. *Andrographis paniculata* is one of the important medicinal species in Thailand according to its use value (UV) (Phumthum et al., 2018). Although the plant is used by both ethnic groups in completely different ways (Figure 3), it provides a tremendous information on treatments of mild COVID-19 symptoms and it has antiviral activities, especially for coronaviruses (Shi et al., 2020; Bhattacharya et al., 2021; Jiang et al., 2021). Other plants, such as *Mimosa pigra* which is used for treatment of fever by the Karen, *Hedyotis acutangular* and *Pseudelephantopus spicatus* which are used to treat cough by the Hmong, and *Cassia fistula* which is used to treat diarrhea by the Hmong, are interesting candidates because of their abundant uses in treatments of specific symptoms. However, pharmacological support for using these plants for treatments is rare. Further studies should focus on phytochemicals and biological activities of these plants used for treatments of the specified symptoms.

Thai Hmong and Karen traditional knowledge on ethnomedicinal plant uses provides interesting information on medicinal plant candidates for treatments of the mild COVID-19 symptoms. Several of the proposed species have substantial additional scientific evidences to support the efficacy of the traditional uses while others need more studies. However, the COVID-19 is a new deadly emerging disease. Moreover, some infected patients would have complex symptoms or serious symptoms. Here, we strongly emphasize that using these medicinal plants for treatments of the mild COVID-19 symptoms must be under strict medical supervision.

We propose several plant species for treatments of mild COVID-19 symptoms. In the worst-case scenario, such as we are currently experiencing in which we may have to stay with the disease for an unknown period of time, using these plants as an alternative choice of treatments would be suitable for many people, especially in developing countries. However, if it turns out that the vaccines have no strong side effects and are efficient to activate our immune system to protect us from the disease for a long time, we may not need to rely on these proposed medicinal plants for treatments of the mild symptoms. The traditional knowledge of the Hmong and the Karen documented in this study remains important for the development of medicines based on traditional knowledge. Although COVID-19 may disappear within a few years, fever, cough, diarrhea, muscle pain, and aches, rash on skin, headache, sore throat, and conjunctivitis are common symptoms that the majority of the world population experience annually. Therefore, studying medicines from these proposed plants is necessary and would save and may benefit the world.

## Conclusion

We documented 1230 uses of 491 plants in treatments of mild COVID-19 symptoms as listed by WHO. The plants were mainly used to treat a subset of mild COVID-19 symptoms, including fever, cough, diarrhea, muscle pain, rash, headache, sore throat, and conjunctivitis. Sixty of the species were in common use. *Strobilanthes cusia, Acorus calamus*, *Melicope glomerata*, *Andrographis paniculata*, *Teucrium viscidum*, *Bidens biternata*, *Paederia pilifera*, *Mimosa pigra*, *Monosis parishii*, *Artemisia vulgaris*, were used to treat fever; *Zingiber officinale*, *Blumea balsamifera*, *Elephantopus scaber*, *Andrographis paniculata*, *Teucrium viscidum*, *Pseudelephantopus spicatus*, *Hedyotis acutangula*, *Cyclea barbata*, *Alpinia malaccensis* were used to treat cough; *Psidium guajava*, *Zingiber ottensii*, *Cassia fistula*, and *Aster indicus* were used to treat diarrhea; *Verbena officinalis* and *Dichrocephala integrifolia* were used to treat skin rash; *Artemisia vulgaris* was used to treat headache, and *Pothos scandens* was used to treat muscle pain. Many of these commonly used species had additional scientific evidences that supported their ethnomedicinal uses among the Thai Hmong and Karen. However, because the COVID-19 is a new emerging disease and the symptoms have insufficient clinical data on treatments to support, we recommend that future studies should test the efficacy of these plants on treatments of the symptoms of the COVID-19. We may have to deal with COVID-19 for only a few years in the future. In this case, the plants may not be relevant for treatment of the mild COVID-19 symptoms. However, these symptoms are common and associated with many common negative health conditions. Scientific experiments need to be done to confirm their efficacy and safety. Finally, we hope that traditional knowledge from these ethnic groups would alleviate suffering of patients who have these symptoms, especially in rural communities whos lives rely on medicinal plants for their primary health care.

## Data Availability

The original contributions presented in the study are included in the article/Supplementary Material, further inquiries can be directed to the corresponding author.
