# Supplementary material for: Medicinal Plants Used for Treating Mild Covid-19 Symptoms Among Thai Karen and Hmong
Source: Front Pharmacol. 2021 Jul 20;12:699897. doi: 10.3389/fphar.2021.699897 (PMC8329454; doi:10.3389/fphar.2021.699897)
Supplement: Supplementary file 1 [file Table1.DOCX]

**Supplementary Materials**

Medicinal plants used for treating mild Covid-19 symptoms among Thai Karen and Hmong

Methee Phumthum, Varangrat Nguanchoo, Henrik Balslev

**Table S1** List of species, the numbers of use reports, and use values of ethnomedicinal plants used by Thai Karen and Hmong for treatment of mild COVID-19 symptoms

| **Family/species name** | **No. of use reports** | **UV** | **Source** | **Voucher number** |
| --- | --- | --- | --- | --- |
| **Acanthaceae** |  |  |  |  |
| *Andrographis paniculata* (Burm.f.) Nees | 9 | 0.16 | Field study  Junkhonkaen, 2012;  Pongamornkul, 2003;  Srithi, 2012  Tangjitman, 2014; | 712 |
| *Clinacanthus nutans* (Burm.f.) Lindau | 1 | 0.02 | Sukkho, 2008 |  |
| *Dicliptera chinensis* (L.) Juss. | 2 | 0.04 | Anderson, 1993;  Srithi, 2012 |  |
| *Hypoestes phyllostachya* Baker | 1 | 0.02 | Trisonthi and Trisonthi, 1995 |  |
| *Justicia gendarussa* Burm.f. | 5 | 0.09 | Kantasrila, 2016;  Srithi, 2012;  Tangjitman, 2016 |  |
| *Lepidagathis incurva* Buch.-Ham. ex D.Don | 1 | 0.02 | Srithi, 2012 |  |
| *Phlogacanthus curviflorus* (Nees) Nees | 4 | 0.07 | Field study  Anderson, 1993; | 693 |
| *Rhinacanthus nasutus* (L.) Kurz | 1 | 0.02 | Nuammee, 2012; |  |
| *Ruellia tuberosa* L. | 1 | 0.02 | Srithi, 2012 |  |
| *Sanchezia oblonga* Ruiz & Pav. | 4 | 0.07 | Field study  Imchan, 2006;  Srithi, 2012 | 879, 930 |
| *Strobilanthes cusia* (Nees) Kuntze | 21 | 0.38 | Field study  Pipitkul, 2001;  Srithi, 2012;  Sukkho, 2008;  Tangjitman, 2014 | 613, 871 |
| *Thunbergia coccinea* Wall. ex D.Don | 1 | 0.02 | Kaewdangsai, 2017 |  |
| *Thunbergia laurifolia* Lindl. | 6 | 0.11 | Field study  Kantasrila, 2016;  Kaewdangsai, 2017;  Pongamornkul, 2003;  Yarnvudhi et al., 2016 | 603, 867 |
| **Acoraceae** |  |  |  |  |
| *Acorus calamus* L. | 11 | 0.20 | Field study  Kamwong, 2010;  Kantasrila, 2016;  Pongamornkul, 2003;  Srisanga, 1993;  Srithi, 2012;  Tangjitman, 2014;  Tovaranonte, 1998;  Trisonthi and Trisonthi, 1995 | 764 |
| *Acorus gramineus* Aiton | 3 | 0.05 | Field study  Imchan, 2006 | 705 |
| **Actinidiaceae** |  |  |  |  |
| *Saurauia napaulensis* DC. | 3 | 0.05 | Pongamornkul, 2003;  Tangjitman, 2014 |  |
| *Saurauia roxburghii* Wall. | 1 | 0.02 | Anderson, 1993 |  |
| **Amaranthaceae** |  |  |  |  |
| *Achyranthes bidentata* Blume | 1 | 0.02 | Field study | 746, 824 |
| *Alternanthera bettzickiana* (Regel) G.Nicholson | 2 | 0.04 | Field study | 747, 883 |
| *Alternanthera brasiliana* (L.) Kuntze | 1 | 0.02 | Field study | 633 |
| *Celosia argentea* L. | 1 | 0.02 | Anderson, 1993 |  |
| **Amaryllidaceae** |  |  |  |  |
| *Allium ascalonicum* L. | 1 | 0.02 | Sonnsupub, 2010 |  |
| *Allium sativum* L. | 1 | 0.02 | Sonnsupub, 2010 |  |
| *Crinum × amabile* Donn ex Ker Gawl. | 1 | 0.02 | Srithi, 2012 |  |
| *Crinum asiaticum* L. | 1 | 0.02 | Pongamornkul, 2003 |  |
| *Hippeastrum x johnsonii* Bury | 2 | 0.04 | Field study | - |
| *Scadoxus multiflorus* (Martyn) Raf. | 1 | 0.02 | Sukkho, 2008 |  |
| *Zephyranthes carinata* Herb. | 1 | 0.02 | Field study | 819, 828 |
| *Zephyranthes rosea* Lindl. | 3 | 0.05 | Pongamornkul, 2003;  Srisanga, 1993;  Trisonthi and Trisonthi, 2009 |  |
| **Anacardiaceae** |  |  |  |  |
| *Anacardium occidentale* L. | 1 | 0.02 | Sonnsupub, 2010 |  |
| *Rhus chinensis* Mill. | 2 | 0.04 | Anderson, 1993 |  |
| *Spondias pinnata* (L.f.) Kurz | 3 | 0.05 | Anderson, 1993;  Tangjitman, 2014;  Trisonthi and Trisonthi, 1995 |  |
| **Annonaceae** |  |  |  |  |
| *Desmos dumosus* (Roxb.) Saff. | 1 | 0.02 | Junsongduang, 2014 |  |
| *Desmos macrocarpus* Bân | 2 | 0.04 | Kaewdangsai, 2017 |  |
| *Miliusa thorelii Finet* & Gagnep. | 1 | 0.02 | Tangjitman, 2014 |  |
| **Apiaceae** |  |  |  |  |
| *Anethum graveolens* L. | 2 | 0.04 | Field study | 676 |
| *Centella asiatica* (L.) Urb. | 6 | 0.11 | Field study  Anderson, 1993;  Kantasrila, 2016  Sukkho, 2008;  Winijchaiyanan, 1995 | 979, 1057 |
| *Coriandrum sativum* L. | 2 | 0.04 | Nuammee, 2012;  Srithi, 2012 |  |
| *Eryngium foetidum* L. | 1 | 0.02 | Field study | 711 |
| *Foeniculum vulgare* Mill. | 1 | 0.02 | Anderson, 1993 |  |
| *Oenanthe javanica* (Blume) DC. | 2 | 0.04 | Anderson, 1993;  Kaewdangsai, 2017 |  |
| **Apocynaceae** |  |  |  |  |
| *Alstonia rostrata* C.E.C.Fisch. | 3 | 0.05 | Kaewdangsai, 2017;  Kantasrila, 2016 |  |
| *Alstonia scholaris* (L.) R.Br. | 6 | 0.11 | Junsongduang, 2014  Trisonthi and Trisonthi, 1995;  Trisonthi and Trisonthi, 2009;  Sukkho, 2008;  Tangjitman, 2016;  Winijchaiyanan, 1995 |  |
| *Amalocalyx microlobus* Pierre ex Spire | 1 | 0.02 | Trisonthi and Trisonthi, 1995 |  |
| *Amphineurion marginatum* (Roxb.) D.J.Middleton | 1 | 0.02 | Sukkho, 2008 |  |
| *Anodendron parviflorum* (Roxb.) I.M.Turner | 1 | 0.02 | Field study | 1017 |
| *Calotropis gigantea* (L.) W.T.Aiton | 1 | 0.02 | Junkhonkaen, 2012 |  |
| *Holarrhena pubescens* Wall. ex G.Don | 1 | 0.02 | Anderson, 1993 |  |
| *Kopsia arborea* Blume | 1 | 0.02 | Tangjitman, 2014 |  |
| *Plumeria obtusa* L. | 3 | 0.05 | Junkhonkaen, 2012;  Kantasrila, 2016 |  |
| *Plumeria rubra* L. | 1 | 0.02 | Kaewdangsai, 2017 |  |
| *Rauvolfia verticillata* (Lour.) Baill. | 1 | 0.02 | Tangjitman, 2016 |  |
| *Streptocaulon juventas* (Lour.) Merr. | 1 | 0.02 | Anderson, 1993 |  |
| *Tabernaemontana pandacaqui* Poir. | 1 | 0.02 | Srithi, 2012 |  |
| *Wattakaka volubilis* (L.f.) Stapf | 1 | 0.02 | Anderson, 1993 |  |
| *Wrightia religiosa* (Teijsm. & Binn.) Benth. ex Kurz | 1 | 0.02 | Nuammee, 2012 |  |
| **Araceae** |  |  |  |  |
| *Alocasia cucullata* (Lour.) G.Don | 4 | 0.07 | Anderson, 1993;  Srithi, 2012 |  |
| *Alocasia navicularis* (K.Koch & C.D.Bouché) K.Koch & C.D.Bouché | 1 | 0.02 | Field study | 846 |
| *Leucocasia gigantea* (Blume) Schott | 1 | 0.02 | Tovaranonte, 1998 |  |
| *Pothos chinensis* (Raf.) Merr. | 8 | 0.14 | Anderson, 1993;  Imchan, 2006;  Kaewdangsai, 2017;  Kantasrila, 2016;  Srithi, 2012 |  |
| *Pothos scandens* L. | 7 | 0.13 | Sukkho, 2008;  Tangjitman, 2014 |  |
| *Typhonium trilobatum* (L.) Schott | 2 | 0.04 | Anderson, 1993 |  |
| **Araliaceae** |  |  |  |  |
| *Hydrocotyle sibthorpioides* Lam. | 2 | 0.04 | Srithi, 2012 |  |
| *Schefflera leucantha* R.Vig. | 1 | 0.02 | Sukkho, 2008 |  |
| *Schefflera venulosa* (Wight & Arn.) Harms | 1 | 0.02 | Winijchaiyanan, 1995 |  |
| **Arecaceae** |  |  |  |  |
| *Areca catechu* L. | 1 | 0.02 | Tangjitman, 2014 |  |
| *Arenga pinnata* (Wurmb) Merr. | 1 | 0.02 | Anderson, 1993 |  |
| **Asparagaceae** |  |  |  |  |
| *Asparagus filicinus* Buch.-Ham. ex D.Don | 2 | 0.04 | Anderson, 1993;  Srithi, 2012 |  |
| *Aspidistra elatior* Blume | 1 | 0.02 | Klibai, 2013 |  |
| *Chlorophytum nepalense* (Lindl.) Baker | 1 | 0.02 | Srithi, 2012 |  |
| *Dracaena roxburghiana* (Schult. & Schult.f.) Byng & Christenh. | 1 | 0.02 | Anderson, 1993 |  |
| *Dracaena terniflora* Roxb. | 1 | 0.02 | Srithi, 2012 |  |
| *Dracaena trifasciata* (Prain) Mabb. | 1 | 0.02 | Kaewdangsai, 2017 |  |
| *Chlorophytum nepalense* (Lindl.) Baker | 1 | 0.02 | Anderson, 1993 |  |
| *Peliosanthes caesia* J.M.H.Shaw | 2 | 0.04 | Kantasrila, 2016 |  |
| *Peliosanthes macrophylla* Wall. ex Baker | 1 | 0.02 | Anderson, 1993 |  |
| *Peliosanthes teta* Andrews | 1 | 0.02 | Anderson, 1993 |  |
| **Asphodelaceae** |  |  |  |  |
| *Aloe vera* (L.) Burm.f. | 1 | 0.02 | Anderson, 1993 |  |
| *Dianella ensifolia* (L.) Redouté | 2 | 0.04 | Field study | 660, 1013 |
| **Aspleniaceae** |  |  |  |  |
| *Asplenium nidus* L. | 1 | 0.02 | Srithi, 2012 |  |
| *Diplazium esculentum* (Retz.) Sw. | 1 | 0.02 | Noitana et al., 2013 |  |
| **Asteraceae** |  |  |  |  |
| *Ageratina adenophora* (Spreng.) R.M.King & H.Rob. | 2 | 0.04 | Kaewdangsai, 2017  Kantasrila, 2016 |  |
| *Ageratum conyzoides* L. | 8 | 0.14 | Field study  Anderson, 1993;  Sukkho, 2008  Tovaranonte, 1998; | 951, 974 |
| *Artemisia atrovirens* Hand.-Mazz. | 2 | 0.04 | Anderson, 1993 |  |
| *Artemisia lactiflora* Wall. ex DC. | 1 | 0.02 | Field study | 785, 916 |
| *Artemisia pallens* Wall. ex DC. | 2 | 0.04 | Trisonthi and trisonthi, 2009 |  |
| *Artemisia verlotiorum* Lamotte | 7 | 0.13 | Srithi, 2012 |  |
| *Artemisia vulgaris* L. | 10 | 0.18 | Field study  Srithi, 2012 | 680 |
| *Aster indicus* L. | 9 | 0.16 | Field study  Srithi, 2012 | 752, 877 |
| *Bidens biternata* (Lour.) Merr. & Sherff | 8 | 0.14 | Field study  Tovaranonte, 1998 | 645 |
| *Bidens pilosa* L. | 5 | 0.09 | Anderson, 1993;  Tichachart, 2004 |  |
| *Blumea balsamifera* (L.) DC. | 17 | 0.30 | Field study  Anderson, 1993;  Imchan, 2006  Kaewdangsai, 2017;  Kantasrila, 2016  Srithi, 2012;  Tovaranonte, 1998 | 795 |
| *Blumea fistulosa* (Roxb.) Kurz | 1 | 0.02 | Anderson, 1993 |  |
| *Blumea flava* DC. | 3 | 0.05 | Field study | 938 |
| *Blumea lacera* (Burm.f.) DC. | 1 | 0.02 | Anderson, 1993 |  |
| *Blumea lanceolaria* (Roxb.) Druce | 1 | 0.02 | Srithi, 2012 |  |
| *Chromolaena odorata* (L.) R.M.King & H.Rob. | 9 | 0.16 | Field study  Anderson, 1993;  Kaewdangsai, 2017;  Kantasrila, 2016; | 624, 970 |
| *Crassocephalum crepidioides* (Benth.) S.Moore | 1 | 0.02 | Kaewdangsai, 2017 |  |
| *Cyanthillium cinereum* (L.) H.Rob. | 3 | 0.05 | Junkhonkaen, 2012;  Srithi, 2012 |  |
| *Dichrocephala integrifolia* (L.f.) Kuntze | 8 | 0.14 | Field study  Moonjai, 2017;  Srithi, 2012;  Tovaranonte, 1998 | 606, 791 |
| *Duhaldea cappa* (Buch.-Ham. ex D.Don) Pruski & Anderb. | 12 | 0.21 | Field study  Junsongduang, 2014;  Srithi, 2012;  Sukkho, 2008 | 695, 939 |
| *Elephantopus scaber* L. | 15 | 0.27 | Field study  Anderson, 1993;  Kaewdangsai, 2017;  Kantasrila, 2016;  Srithi, 2012;  Sukkho, 2008;  Trisonthi and trisonthi, 2009;  Winijchaiyanan, 1995 | 640, 757 |
| *Ethulia conyzoides* subsp. *conyzoides* | 1 | 0.02 | Imchan, 2006 |  |
| *Eupatorium fortunei* Turcz. | 1 | 0.02 | Prachuabaree, 2008 |  |
| *Gynura nepalensis* DC. | 1 | 0.02 | Srithi, 2012 |  |
| *Laggera crispata* (Vahl) Hepper & J.R.I.Wood | 2 | 0.04 | Field study | 614 |
| *Microglossa pyrifolia* (Lam.) Kuntze | 2 | 0.04 | Field study | 1000 |
| *Monosis parishii* (Hook.f.) H.Rob. & Skvarla | 5 | 0.09 | Field study | 756, 968 |
| *Monosis volkameriifolia* (DC.) H.Rob. & Skvarla | 1 | 0.02 | Tangjitman, 2016 |  |
| *Pseudelephantopus spicatus* (Juss. ex Aubl.) C.F.Baker | 6 | 0.11 | Field study | 775, 812 |
| *Sphagneticola calendulacea* (L.) Pruski | 1 | 0.02 | Tovaranonte, 1998 |  |
| *Tithonia diversifolia* (Hemsl.) A.Gray | 3 | 0.05 | Kantasrila, 2016;  Srithi, 2012;  Yarnvudhi et al., 2016 |  |
| **Balsaminaceae** |  |  |  |  |
| *Impatiens balsamina* L. | 2 | 0.04 | Field study  Srithi, 2012 | 912 |
| **Berberidaceae** |  |  |  |  |
| *Berberis napaulensis* (DC.) Spreng. | 1 | 0.02 | Tangjitman, 2016 |  |
| **Betulaceae** |  |  |  |  |
| *Betula alnoides* Buch.-Ham. ex D.Don | 2 | 0.04 | Kantasrila, 2016;  Trisonthi and Trisonthi, 1995 |  |
| **Bignoniacea** |  |  |  |  |
| *Markhamia stipulata* (Wall.) Seem. | 1 | 0.02 | Anderson, 1993 |  |
| *Mayodendron igneum* (Kurz) Kurz | 1 | 0.02 | Pongamornkul,2003 |  |
| *Millingtonia hortensis* L.f. | 2 | 0.04 | Anderson, 1993 |  |
| *Nyctocalos brunfelsiiflorum* Teijsm. & Binn. | 1 | 0.02 | Kantasrila, 2016 |  |
| *Oroxylum indicum* (L.) Kurz | 4 | 0.07 | Field study  Anderson, 1993 | 1039 |
| **Boraginaceae** |  |  |  |  |
| *Heliotropium indicum* L. | 1 | 0.02 | Tangjitman, 2014 |  |
| **Bromeliaceae** |  |  |  |  |
| *Ananas comosus* (L.) Merr. | 1 | 0.02 | Srisanga, 1993 |  |
| **Burseraceae** |  |  |  |  |
| *Garuga pinnata* Roxb. | 1 | 0.02 | Trisonthi and Trisonthi, 1995 |  |
| **Buxaceae** |  |  |  |  |
| *Buxus cochinchinensis* Pierre ex Gagnep. | 1 | 0.02 | Prachuabaree, 2008 |  |
| **Campanulaceae** |  |  |  |  |
| *Cyclocodon lancifolius* (Roxb.) Kurz | 1 | 0.02 | Kantasrila, 2016 |  |
| **Cannabaceae** |  |  |  |  |
| *Celtis tetrandra* Roxb. | 6 | 0.11 | Kaewdangsai, 2017;  Kantasrila, 2016 |  |
| **Caprifoliaceae** |  |  |  |  |
| *Valeriana hardwickei* Wall. | 1 | 0.02 | Srithi, 2012 |  |
| **Caricaceae** |  |  |  |  |
| *Carica papaya* L. | 2 | 0.04 | Srithi, 2012 |  |
| **Caryophyllaceae** |  |  |  |  |
| *Drymaria diandra* Blume | 1 | 0.02 | Srithi, 2012 |  |
| **Celastraceae** |  |  |  |  |
| *Celastrus paniculatus* Willd. | 3 | 0.05 | Kamwong, 2010;  Moonjai, 2017;  Tangjitman, 2014 |  |
| *Salacia verrucosa* Wight | 1 | 0.02 | Kantasrila, 2016 |  |
| **Chloranthaceae** |  |  |  |  |
| *Chloranthus elatior* Link | 9 | 0.16 | Field study  Anderson, 1993;  Srithi, 2012; | 809, 1059 |
| *Chloranthus nervosus* Collett & Hemsl. | 2 | 0.04 | Field study | 1036 |
| *Sarcandra glabra* (Thunb.) Nakai | 2 | 0.04 | Kantasrila, 2016 |  |
| **Clusiaceae** |  |  |  |  |
| *Calophyllum polyanthum* Wall. ex Choisy | 1 | 0.02 | Junsongduang, 2014 |  |
| *Garcinia xanthochymus* Hook. f. ex. T. Anderson | 1 | 0.02 | Pongamornkul, 2003 |  |
| **Colchicaceae** |  |  |  |  |
| *Disporum calcaratum* D.Don | 1 | 0.02 | Tovaranonte, 1998 |  |
| **Combretaceae** |  |  |  |  |
| *Anogeissus acuminata* (Roxb. ex DC.) Wall. ex Guill. & Perr. | 3 | 0.05 | Pongamornkul, 2003;  Puling, 2001 |  |
| *Combretum deciduum* Collett & Hemsl. | 1 | 0.02 | Anderson, 1993 |  |
| *Combretum indicum* (L.) DeFilipps | 1 | 0.02 | Tangjitman, 2016 |  |
| *Getonia floribunda* Roxb. | 1 | 0.02 | Mahawongsanan, 2008 |  |
| *Terminalia bellirica* (Gaertn.) Roxb. | 1 | 0.02 | Pongamornkul, 2003 |  |
| *Terminalia chebula* Retz. | 3 | 0.05 | Junkhonkaen, 2012;  Junsongduang, 2014;  Pongamornkul, 2003 |  |
| **Commelinaceae** |  |  |  |  |
| *Callisia repens* (Jacq.) L. | 2 | 0.04 | Srithi, 2012 |  |
| *Tradescantia spathacea* Sw. | 1 | 0.02 | Junkhonkaen, 2012 |  |
| *Tradescantia zebrina* Bosse | 2 | 0.04 | Field study  Anderson, 1993 | 799, 959 |
| **Connaraceae** |  |  |  |  |
| *Cnestis palala* (Lour.) Merr. | 1 | 0.02 | Kantasrila, 2016 |  |
| **Convolvulaceae** |  |  |  |  |
| *Cuscuta chinensis* Lam. | 2 | 0.04 | Kaewdangsai, 2017 |  |
| *Cuscuta japonica* Choisy | 1 | 0.02 | Nuammee, 2012 |  |
| *Cuscuta reflexa* Roxb. | 1 | 0.02 | Anderson, 1993 |  |
| *Ipomoea batatas* (L.) Lam. | 1 | 0.02 | Tangjitman, 2016 |  |
| *Merremia hirta* (L.) Merr. | 1 | 0.02 | Field study | 623 |
| **Cornaceae** |  |  |  |  |
| *Alangium uniloculare* (Griff.) King | 1 | 0.02 | Tovaranonte, 1998 |  |
| *Hellenia speciosa* (J.Koenig) S.R.Dutta | 2 | 0.04 | Field study  Anderson, 1993 | 969, 1006 |
| **Crassulaceae** |  |  |  |  |
| *Kalanchoe laciniata* (L.) DC. | 1 | 0.02 | Srithi, 2012 |  |
| *Kalanchoe pinnata* (Lam.) Pers. | 2 | 0.04 | Srithi, 2012 |  |
| *Sedum sarmentosum* Bunge | 2 | 0.04 | Srithi, 2012 |  |
| **Cucurbitaceae** |  |  |  |  |
| *Coccinia grandis* (L.) Voigt | 1 | 0.02 | Gunsuwan, 2011 |  |
| *Hodgsonia macrocarpa* (Blume) Cogn. | 1 | 0.02 | Srithi, 2012 |  |
| *Momordica charantia* L. | 1 | 0.02 | Kantasrila, 2016 |  |
| **Cyperaceae** |  |  |  |  |
| *Cyperus brevifolius* (Rottb.) Hassk. | 1 | 0.02 | Chaunchom, 2011 |  |
| *Cyperus mindorensis* (Steud.) Huygh | 1 | 0.02 | Srithi, 2012 |  |
| **Dioscoreaceae** |  |  |  |  |
| *Dioscorea bulbifera* L. | 1 | 0.02 | Srithi, 2012 |  |
| *Tacca chantrieri* André | 6 | 0.11 | Field study  Anderson, 1993 | 690, 810 |
| *Tacca integrifolia* Ker Gawl. | 1 | 0.02 | Srisanga, 1993 |  |
| **Dipterocarpaceae** |  |  |  |  |
| *Dipterocarpus obtusifolius* Teijsm. ex Miq. | 1 | 0.02 | Tangjitman, 2014 |  |
| *Hopea helferi* (Dyer) Brandis | 1 | 0.02 | Moonjai, 2017 |  |
| **Ebenaceae** |  |  |  |  |
| *Diospyros areolata* King & Gamble | 1 | 0.02 | Prachuabaree, 2008 |  |
| *Diospyros castanea* (Craib) H.R.Fletcher | 1 | 0.02 | Junsongduang, 2014 |  |
| **Equisetaceae** |  |  |  |  |
| *Hippochaete debilis* (Roxb. ex Vaucher) Holub | 1 | 0.02 | Kantasrila, 2016 |  |
| **Ericaceae** |  |  |  |  |
| *Agapetes hosseana* Diels | 1 | 0.02 | Tangjitman, 2016 |  |
| **Euphorbiaceae** |  |  |  |  |
| *Acalypha spiciflora* Burm.f. | 1 | 0.02 | Anderson, 1993 |  |
| *Baliospermum calycinum* Müll.Arg. | 1 | 0.02 | Winijchaiyanan, 1995 |  |
| *Baliospermum solanifolium* (Burm.) Suresh | 1 | 0.02 | Sonnsupub, 2010 |  |
| *Chrozophora tinctoria* (L.) A.Juss. | 1 | 0.02 | Winijchaiyanan, 1995 |  |
| *Cleidion javanicum* Blume | 3 | 0.05 | Kamwong, 2010;  Pongamornkul, 2003;  Trisonthi and Trisonthi, 1995 |  |
| *Codiaeum variegatum* (L.) Rumph. ex A.Juss. | 1 | 0.02 | Nuammee, 2012 |  |
| *Croton sepalinus* Airy Shaw | 1 | 0.02 | Prachuabaree, 2008 |  |
| *Euphorbia heterophylla* L. | 1 | 0.02 | Kantasrila, 2016 |  |
| *Falconeria insignis* Royle | 3 | 0.05 | Pongamornkul, 2003 |  |
| *Homonoia riparia* Lour. | 1 | 0.02 | Nuammee, 2012 |  |
| *Jatropha curcas* L. | 2 | 0.04 | Field study  Winijchaiyanan, 1995 | 781, 921 |
| *Jatropha podagrica* Hook. | 1 | 0.02 | Tangjitman, 2016 |  |
| *Macaranga triloba* (Thunb.) Müll.Arg. | 1 | 0.02 | Srithi, 2012 |  |
| *Mallotus philippensis* (Lam.) Müll.Arg. | 2 | 0.04 | Kantasrila, 2016 |  |
| *Manihot esculenta* Crantz | 1 | 0.02 | Field study | 955 |
| *Ricinus communis* L. | 7 | 0.13 | Anderson, 1993;  Srisanga, 1993;  Srithi, 2012;  Tichachart, 2004;  Winijchaiyanan, 1995 |  |
| *Triadica cochinchinensis* Lour. | 1 | 0.02 | Junsongduang, 2014 |  |
| **Fabaceae** |  |  |  |  |
| *Acrocarpus fraxinifolius* Wight & Arn. | 1 | 0.02 | Junsongduang, 2014 |  |
| *Adenanthera pavonina* L. | 1 | 0.02 | Tichachart, 2004 |  |
| *Aeschynomene americana* L. | 1 | 0.02 | Sutjaritjai et al., 2019 |  |
| *Albizia lebbeck* (L.) Benth. | 2 | 0.04 | Srithi, 2012 |  |
| *Albizia procera* (Roxb.) Benth. | 1 | 0.02 | Sutjaritjai et al., 2019 |  |
| *Archidendron clypearia* (Jack) I.C.Nielsen | 3 | 0.05 | Tovaranonte, 1998;  Trisonthi and Trisonthi, 2009;  Winijchaiyanan, 1995 |  |
| *Bauhinia ornata* Kurz | 1 | 0.02 | Sutjaritjai et al., 2019 |  |
| *Biancaea sappan* (L.) Tod. | 12 | 0.21 | Field study  Imchan, 2006;  Junkhonkaen, 2012;  Klibai, 2013;  Sukkho, 2008 | - |
| *Cassia fistula* L. | 5 | 0.09 | Field study  Nuammee, 2012; | - |
| *Crotalaria breviflora* DC. | 2 | 0.04 | Sutjaritjai et al., 2019 |  |
| *Dalbergia cana* Graham ex Kurz | 1 | 0.02 | Junsongduang, 2014 |  |
| *Dalbergia cultrata* T.S.Ralph | 2 | 0.04 | Sutjaritjai et al., 2019;  Tangjitman, 2016 |  |
| *Dalbergia ovata* Graham ex Benth. | 1 | 0.02 | Sukkho, 2008 |  |
| *Dalbergia retusa* Hemsl. | 1 | 0.02 | Junsongduang, 2014 |  |
| *Dalbergia stipulacea* Roxb. | 1 | 0.02 | Kantasrila, 2016 |  |
| *Derris elliptica* (Wall.) Benth. | 4 | 0.07 | Kaewdangsai, 2017;  Kantasrila, 2016;  Sutjaritjai et al., 2019 |  |
| *Desmodium velutinum* (Willd.) DC. | 1 | 0.02 | Sutjaritjai et al., 2019 |  |
| *Entada glandulosa* Pierre ex Gagnep. | 2 | 0.04 | Kantasrila, 2016;  Srithi, 2012 |  |
| *Eriosema chinense* Vogel | 1 | 0.02 | Sutjaritjai et al., 2019 |  |
| *Flemingia stricta* Roxb. | 1 | 0.02 | Field study | 635, 642 |
| *Grona heterocarpos* (L.) H.Ohashi & K.Ohashi | 1 | 0.02 | Anderson, 1993 |  |
| *Indigofera caloneura* Kurz | 1 | 0.02 | Sutjaritjai et al., 2019 |  |
| *Indigofera hendecaphylla* Jacq. | 1 | 0.02 | Sutjaritjai et al., 2019 |  |
| *Indigofera tinctoria* L. | 1 | 0.02 | Sutjaritjai et al., 2019 |  |
| *Leucaena leucocephala* (Lam.) de Wit | 1 | 0.02 | Tichachart, 2004 |  |
| *Mimosa diplotricha* C.Wright | 1 | 0.02 | Sutjaritjai et al., 2019 |  |
| *Mimosa pigra* L. | 6 | 0.11 | Junkhonkaen, 2012;  Kaewdangsai, 2017;  Kantasrila, 2016;  Sutjaritjai et al., 2019;  Winijchaiyanan, 1995 |  |
| *Mimosa pudica* L. | 2 | 0.04 | Field study  Srithi, 2012 | 602, 745 |
| *Psophocarpus tetragonolobus* (L.) DC. | 1 | 0.02 | Srithi, 2012 |  |
| *Senegalia catechu* (L.f.) P.J.H.Hurter & Mabb. | 1 | 0.02 | Sutjaritjai et al., 2019 |  |
| *Senegalia rugata* (Lam.) Britton & Rose | 3 | 0.05 | Noitana et al., 2013;  Winijchaiyanan, 1995 |  |
| *Senna alata* (L.) Roxb. | 1 | 0.02 | Sutjaritjai et al., 2019 |  |
| *Senna hirsuta* (L.) H.S.Irwin & Barneby | 1 | 0.02 | Sutjaritjai et al., 2019 |  |
| *Senna occidentalis* (L.) Link | 1 | 0.02 | Sutjaritjai et al., 2019 |  |
| *Senna tora* (L.) Roxb. | 2 | 0.04 | Pongamornkul, 2003 |  |
| *Tadehagi triquetrum* (L.) H.Ohashi | 8 | 0.14 | Field study  Anderson, 1993;  Kaewdangsai, 2017;  Kantasrila, 2016;  Sutjaritjai et al., 2019 | 936 |
| *Tamarindus indica* L. | 3 | 0.05 | Noitana et al., 2013;  Sutjaritjai et al., 2019 |  |
| **Gentianaceae** |  |  |  |  |
| *Canscora andrographioides* Griff. ex C.B.Clarke | 3 | 0.05 | Winijchaiyanan, 1995 |  |
| *Exacum pteranthum* Wall. ex G.Don | 1 | 0.02 | Field study | 944 |
| **Gnetaceae** |  |  |  |  |
| *Gnetum montanum* Markgr. | 3 | 0.05 | Mahawongsanan, 2008;  Puling, 2001 |  |
| **Hernandiaceae** |  |  |  |  |
| *Illigera trifoliata* (Griff.) Dunn | 2 | 0.04 | Kantasrila, 2016 |  |
| **Hypericaceae** |  |  |  |  |
| *Cratoxylum formosum* (Jack) Benth. & Hook. f. ex Dyer | 3 | 0.05 | Kamwong, 2010;  Mahawongsanan, 2008;  Puling, 2001 |  |
| *Cratoxylum formosum* subsp. *formosum* | 1 | 0.02 | Srithi, 2012 |  |
| **Hypoxidaceae** |  |  |  |  |
| *Curculigo capitulata* (Lour.) Kuntze | 2 | 0.04 | Kantasrila, 2016;  Srithi, 2012 |  |
| *Curculigo latifolia* Dryand. ex W.T.Aiton | 2 | 0.04 | Field study | 692, 638 |
| **Iridaceae** |  |  |  |  |
| *Eleutherine bulbosa* (Mill.) Urb. | 6 | 0.11 | Field study  Anderson, 1993;  Kamwong, 2010;  Pongamornkul, 2003;  Srithi, 2012;  Trisonthi and Trisonthi, 1995 | - |
| *Gladiolus hortulanus* L.H. Bailey | 3 | 0.05 | Field study | 792, 815 |
| *Iris domestica* (L.) Goldblatt & Mabb. | 2 | 0.04 | Nuammee, 2012;  Pongamornkul, 2003 |  |
| *Trimezia steyermarkii* R.C.Foster | 1 | 0.02 | Field study | 818 |
| **Juglandaceae** |  |  |  |  |
| *Engelhardia spicata* Lechen ex Blume | 1 | 0.02 | Kamwong, 2010 |  |
| **Lamiaceae** |  |  |  |  |
| *Callicarpa arborea* Roxb. | 1 | 0.02 | Junsongduang, 2014 |  |
| *Callicarpa rubella* Lindl. | 1 | 0.02 | Junsongduang, 2014 |  |
| *Clerodendrum colebrookeanum* Walp. | 1 | 0.02 | Nuammee, 2012 |  |
| *Clerodendrum infortunatum* L. | 1 | 0.02 | Junkhonkaen, 2012 |  |
| *Clerodendrum japonicum* (Thunb.) Sweet | 1 | 0.02 | Anderson, 1993 |  |
| *Clerodendrum nutans* Wall. ex Jack | 2 | 0.04 | Kantasrila, 2016 |  |
| *Clerodendrum paniculatum* L. | 1 | 0.02 | Sukkho, 2008 |  |
| *Coleus amboinicus* Lour. | 2 | 0.04 | Sukkho, 2008 |  |
| *Congea tomentosa* Roxb. | 3 | 0.05 | Anderson, 1993;  Pongamornkul, 2003 |  |
| *Elsholtzia penduliflora* W.W.Sm. | 1 | 0.02 | Srithi, 2012 |  |
| *Glechoma hederacea* L. | 1 | 0.02 | Srithi, 2012 |  |
| *Gmelina arborea* Roxb. ex Sm. | 3 | 0.05 | Kaewdangsai, 2017;  Kantasrila, 2016;  Trisonthi and Trisonthi, 2009 |  |
| *Gomphostemma strobilinum* Wall. ex Benth. | 1 | 0.02 | Krueasan, 2000 |  |
| *Leonurus japonicus* Houtt. | 1 | 0.02 | Nuammee, 2012 |  |
| *Melissa officinalis* L. | 3 | 0.05 | Field study | 783 |
| *Mentha × villosa* Huds. | 2 | 0.04 | Gunsuwan, 2011 |  |
| *Mentha arvensis* L. | 1 | 0.02 | Srithi, 2012 |  |
| *Microtoena insuavis* (Hance) Prain ex Briq. | 1 | 0.02 | Tangjitman, 2014 |  |
| *Ocimum basilicum* L. | 2 | 0.04 | Gunsuwan, 2011;  Kantasrila, 2016 |  |
| *Orthosiphon aristatus* (Blume) Miq. | 1 | 0.02 | Srithi, 2012 |  |
| *Perilla frutescens* (L.) Britton | 1 | 0.02 | Imchan, 2006 |  |
| *Rotheca serrata* (L.) Steane & Mabb. | 9 | 0.16 | Anderson, 1993;  Krueasan, 2000;  Kantasrila, 2016;  Sukkho, 2008 |  |
| *Teucrium viscidum* Blume | 12 | 0.21 | Field study  Nuammee, 2012;  Srithi, 2012 | 1052 |
| *Vitex trifolia* L. | 2 | 0.04 | Junkhonkaen, 2012;  Sonnsupub, 2010 |  |
| **Lauraceae** |  |  |  |  |
| *Cassytha filiformis* L. | 4 | 0.07 | Kaewdangsai, 2017;  Sukkho, 2008;  Trisonthi and Trisonthi, 1995 |  |
| *Cinnamomum subavenium* Miq. | 3 | 0.05 | Kantasrila, 2016 |  |
| *Litsea cubeba* (Lour.) Pers. | 3 | 0.05 | Junsongduang, 2014;  Kantasrila, 2016;  Trisonthi and Trisonthi, 1995 |  |
| **Lecythidaceae** |  |  |  |  |
| *Careya arborea* Roxb. | 1 | 0.02 | Anderson, 1993 |  |
| **Liliaceae** |  |  |  |  |
| *Lilium primulinum* Baker | 2 | 0.04 | Sukkho, 2008 |  |
| **Linderniaceae** |  |  |  |  |
| *Bonnaya ruellioides* (Colsm.) Spreng. | 2 | 0.04 | Srithi, 2012 |  |
| *Picria fel-terrae* Lour. | 1 | 0.02 | Field study | 668, 856 |
| *Torenia asiatica* L. | 1 | 0.02 | Srithi, 2012 |  |
| *Torenia crustacea* (L.) Cham. & Schltdl. | 1 | 0.02 | Tovaranonte, 1998 |  |
| **Loranthaceae** |  |  |  |  |
| *Dendrophthoe pentandra* (L.) Miq. | 1 | 0.02 | Kantasrila, 2016 |  |
| *Elytranthe albida* (Blume) Blume | 1 | 0.02 | Kantasrila, 2016 |  |
| *Scurrula ferruginea* (Roxb. ex Jack) Danser | 1 | 0.02 | Kantasrila, 2016 |  |
| *Scurrula parasitica* L. | 2 | 0.04 | Nuammee, 2012;  Tovaranonte, 1998 |  |
| **Magnoliaceae** |  |  |  |  |
| *Magnolia garrettii* (Craib) V.S.Kumar | 1 | 0.02 | Junsongduang, 2014 |  |
| **Malpighiaceae** |  |  |  |  |
| *Hiptage benghalensis* (L.) Kurz | 1 | 0.02 | Winijchaiyanan, 1995 |  |
| *Hiptage candicans* Hook.f. | 1 | 0.02 | Winijchaiyanan, 1995 |  |
| **Malvaceae** |  |  |  |  |
| *Helicteres elongata* Wall. ex Mast. | 4 | 0.07 | Field study  Anderson, 1993;  Tichachart, 2004 | 937, 1037 |
| *Melochia umbellata* (Houtt.) Stapf | 1 | 0.02 | Field study | 630 |
| *Microcos paniculata* L. | 1 | 0.02 | Kantasrila, 2016 |  |
| *Sida acuta* Burm.f. | 4 | 0.07 | Kaewdangsai, 2017  Kantasrila, 2016 |  |
| *Sida cordifolia* L. | 2 | 0.04 | Kantasrila, 2016 |  |
| *Sida rhombifolia* L. | 3 | 0.05 | Kantasrila, 2016  Nuammee, 2012;  Winijchaiyanan, 1995 |  |
| *Triumfetta rhomboidea* Jacq. | 2 | 0.04 | Anderson, 1993;  Nuammee, 2012 |  |
| **Marantaceae** |  |  |  |  |
| *Maranta arundinacea* L. | 2 | 0.04 | Pongamornkul, 2003;  Srithi, 2012 |  |
| *Phrynium pubinerve* Blume | 1 | 0.02 | Anderson, 1993 |  |
| **Marattiaceae** |  |  |  |  |
| *Angiopteris evecta* (G.Forst.) Hoffm. | 2 | 0.04 | Field study  Winijchaiyanan, 1995 | - |
| **Melanthiaceae** |  |  |  |  |
| *Paris polyphylla* Sm. | 1 | 0.02 | Sukkho, 2008 |  |
| **Melastomataceae** |  |  |  |  |
| *Melastoma malabathricum* L. | 2 | 0.04 | Junsongduang, 2014;  Noitana et al., 2013 |  |
| *Melastoma saigonense* (Kuntze) Merr. | 1 | 0.02 | Imchan, 2006 |  |
| *Melastoma sanguineum* Sims | 2 | 0.04 | Kantasrila, 2016 |  |
| *Memecylon pauciflorum* Blume | 1 | 0.02 | Kaewdangsai, 2017 |  |
| *Osbeckia chinensis* L. | 2 | 0.04 | Kantasrila, 2016;  Tangjitman, 2016 |  |
| *Osbeckia stellata* Buch.-Ham. ex D.Don | 1 | 0.02 | Anderson, 1993 |  |
| **Meliaceae** |  |  |  |  |
| *Aglaia lawii* (Wight) C.J.Saldanha | 1 | 0.02 | Junsongduang, 2014 |  |
| *Azadirachta indica* A.Juss. | 2 | 0.04 | Tangjitman, 2016;  Trisonthi and Trisonthi, 1995 |  |
| *Cipadessa baccifera* (Roth) Miq. | 1 | 0.02 | Field study | 767 |
| *Dysoxylum grande* Hiern | 2 | 0.04 | Kantasrila, 2016 |  |
| **Menispermaceae** |  |  |  |  |
| *Cissampelos hispida* Forman | 2 | 0.04 | Anderson, 1993 |  |
| *Cyclea barbata* Miers | 8 | 0.14 | Kantasrila, 2016;  Sukkho, 2008;  Tangjitman, 2014 |  |
| *Pericampylus glaucus* (Lam.) Merr. | 1 | 0.02 | Nuammee, 2012 |  |
| *Tiliacora triandra* (Colebr.) Diels | 1 | 0.02 | Junkhonkaen, 2012 |  |
| *Tinospora baenzigeri* Forman | 1 | 0.02 | Junkhonkaen, 2012 |  |
| *Tinospora crispa* (L.) Hook. f. & Thomson | 7 | 0.13 | Field study  Junkhonkaen, 2012;  Kaewdangsai, 2017;  Sukkho, 2008;  Tangjitman, 2014 | 774 |
| **Moraceae** |  |  |  |  |
| *Artocarpus heterophyllus* Lam. | 2 | 0.04 | Field study | - |
| *Ficus auriculata* Lour. | 2 | 0.04 | Junsongduang, 2014;  Sukkho, 2008 |  |
| *Ficus capillipes* Gagnep. | 1 | 0.02 | Junsongduang, 2014 |  |
| *Ficus elmeri* Merr. | 2 | 0.04 | Anderson, 1993 |  |
| *Ficus hispida* L.f. | 2 | 0.04 | Junkhonkaen, 2012;  Trisonthi and Trisonthi, 1995 |  |
| *Ficus racemosa* L. | 1 | 0.02 | Sonnsupub, 2010 |  |
| *Ficus subulata* Blume | 2 | 0.04 | Srithi, 2012 |  |
| *Maclura cochinchinensis* (Lour.) Corner | 1 | 0.02 | Anderson, 1993 |  |
| *Morus alba* L. | 2 | 0.04 | Field study  Noitana et al., 2013; | 932 |
| *Streblus asper* Lour. | 1 | 0.02 | Prachuabaree, 2008 |  |
| **Musaceae** |  |  |  |  |
| *Ensete glaucum* (Roxb.) Cheesman | 1 | 0.02 | Trisonthi and Trisonthi, 1995 |  |
| *Musa acuminata* Colla | 5 | 0.09 | Anderson, 1993;  Kaewdangsai, 2017 |  |
| *Musa rubra* Wall. ex Kurz | 2 | 0.04 | Field study | - |
| **Myrtaceae** |  |  |  |  |
| *Psidium guajava* L. | 35 | 0.63 | Field study  Anderson, 1993;  Junkhonkaen, 2012;  Kaewdangsai, 2017;  Kantasrila, 2016;  Nuammee, 2012;  Prachuabaree, 2008;  Sonnsupub, 2010;  Srisanga, 1993;  Srithi, 2012;  Tangjitman, 2014;  Tangjitman, 2016;  Tovaranonte, 1998;  Winijchaiyanan, 1995 | 609 |
| *Syzygium cumini* (L.) Skeels | 3 | 0.05 | Junkhonkaen, 2012;  Junsongduang, 2014;  Kantasrila, 2016 |  |
| **Nyctaginacea** |  |  |  |  |
| *Mirabilis jalapa* L. | 1 | 0.02 | Srithi, 2012 |  |
| **Olacaceae** |  |  |  |  |
| *Anacolosa ilicoides* Mast. | 1 | 0.02 | Sukkho, 2008 |  |
| *Jasminum decussatum* Wall. ex G.Don | 1 | 0.02 | Kantasrila, 2016 |  |
| *Jasminum laurifolium* Roxb. ex Hornem. | 2 | 0.04 | Kantasrila, 2016 |  |
| *Jasminum simplicifolium* G.Forst. | 1 | 0.02 | Trisonthi and Trisonthi, 1995 |  |
| **Ophioglossaceae** |  |  |  |  |
| *Ophioglossum costatum* R.Br. | 1 | 0.02 | Anderson, 1993 |  |
| **Orchidaceae** |  |  |  |  |
| *Calanthe cardioglossa* Schltr. | 1 | 0.02 | Klibai, 2013 |  |
| *Strongyleria pannea* (Lindl.) Schuit., Y.P.Ng & H.A.Pedersen | 1 | 0.02 | Field study | 995 |
| **Oxalidaceae** |  |  |  |  |
| *Averrhoa carambola* L. | 2 | 0.04 | Anderson, 1993;  Winijchaiyanan, 1995 |  |
| *Oxalis corniculata* L. | 5 | 0.09 | Srithi, 2012 |  |
| **Pandanaceae** |  |  |  |  |
| *Pandanus furcatus* Roxb. | 1 | 0.02 | Anderson, 1993;  Srithi, 2012;  Tovaranonte, 1998; |  |
| **Papaveraceae** |  |  |  |  |
| *Papaver somniferum* L. | 3 | 0.05 | Anderson, 1993 |  |
| **Passifloraceae** |  |  |  |  |
| *Adenia viridiflora* Craib | 1 | 0.02 | Moonjai, 2017 |  |
| **Pedaliaceae** |  |  |  |  |
| *Sesamum indicum* L. | 1 | 0.02 | Tangjitman, 2016 |  |
| **Phyllanthaceae** |  |  |  |  |
| *Antidesma bunius* (L.) Spreng. | 1 | 0.02 | Winijchaiyanan, 1995 |  |
| *Antidesma ghaesembilla* Gaertn. | 1 | 0.02 | Trisonthi and Trisonthi, 1995 |  |
| *Aporosa villosa* (Lindl.) Baill. | 1 | 0.02 | Tangjitman, 2016 |  |
| *Bischofia javanica* Blume | 4 | 0.07 | Anderson, 1993;  Srithi, 2012;  Tovaranonte, 1998 |  |
| *Breynia quadrangularis* (Willd.) Chakrab. & N.P.Balakr. | 2 | 0.04 | Field study | 628 |
| *Breynia retusa* (Dennst.) Alston | 2 | 0.04 | Srithi, 2012 |  |
| *Flueggea leucopyrus* Willd. | 1 | 0.02 | Kantasrila, 2016 |  |
| *Phyllanthus amarus* Schumach. & Thonn. | 4 | 0.07 | Pongamornkul, 2003;  Prachuabaree, 2008;  Sonnsupub, 2010 |  |
| *Phyllanthus elegans* Wall. ex Müll.Arg. | 1 | 0.02 | Sukkho, 2008 |  |
| *Phyllanthus emblica* L. | 25 | 0.45 | Anderson, 1993;  Krueasan, 2000;  Junkhonkaen, 2012;  Kaewdangsai, 2017;  Kamwong, 2010;  Kantasrila, 2016;  Mahawongsanan, 2008;  Nuammee, 2012;  Prachuabaree, 2008;  Puling, 2001;  Sonnsupub, 2010;  Tangjitman, 2016;  Trisonthi and Trisonthi, 1995 |  |
| *Phyllanthus microcarpus* (Benth.) Müll.Arg. | 5 | 0.09 | Field study | 653 |
| *Phyllanthus niruri* L. | 2 | 0.04 | Srithi, 2012 |  |
| **Piperaceae** |  |  |  |  |
| *Piper betle* L. | 1 | 0.02 | Kantasrila, 2016 |  |
| *Piper interruptum* Opiz | 1 | 0.02 | Kantasrila, 2016 |  |
| *Piper nigrum* L. | 1 | 0.02 | Winijchaiyanan, 1995 |  |
| *Piper retrofractum* Vahl | 2 | 0.04 | Junkhonkaen, 2012;  Winijchaiyanan, 1995 |  |
| *Piper sarmentosum* Roxb. | 4 | 0.07 | Anderson, 1993;  Kamwong, 2010 |  |
| *Zippelia begoniifolia* Blume | 1 | 0.02 | Srithi, 2012 |  |
| **Plantaginaceae** |  |  |  |  |
| *Limnophila aromatica* (Lam.) Merr. | 1 | 0.02 | Kantasrila, 2016 |  |
| *Limnophila rugosa* (Roth) Merr. | 1 | 0.02 | Nuammee, 2012 |  |
| *Plantago major* L. | 22 | 0.39 | Field study  Anderson, 1993;  Imchan, 2006;  Kaewdangsai, 2017;  Kantasrila, 2016;  Nuammee, 2012;  Pongamornkul, 2003;  Srithi, 2012;  Sukkho, 2008;  Winijchaiyanan, 1995 | 737, 825 |
| *Scoparia dulcis* L. | 12 | 0.21 | Srithi, 2012 |  |
| **Plumbaginaceae** |  |  |  |  |
| *Plumbago indica* L. | 2 | 0.04 | Junkhonkaen, 2012;  Kantasrila, 2016 |  |
| *Plumbago zeylanica* L. | 6 | 0.11 | Anderson, 1993;  Imchan, 2006;  Junkhonkaen, 2012;  Srithi, 2012;  Sukkho, 2008 |  |
| **Poaceae** |  |  |  |  |
| *Coix lacryma-jobi* L. | 1 | 0.02 | Sukkho, 2008 |  |
| *Cymbopogon citratus* (DC.) Stapf | 1 | 0.02 | Kaewdangsai, 2017 |  |
| *Dactyloctenium aegyptium* (L.) Willd. | 1 | 0.02 | Junkhonkaen, 2012 |  |
| *Imperata cylindrica* (L.) P.Beauv. | 3 | 0.05 | Kantasrila, 2016 |  |
| *Lophatherum gracile* Brongn. | 1 | 0.02 | Anderson, 1993 |  |
| *Saccharum officinarum* L. | 3 | 0.05 | Nuammee, 2012;  Sukkho, 2008;  Winijchaiyanan, 1995 |  |
| *Schizostachyum pergracile* (Munro) R.B.Majumdar | 1 | 0.02 | Srithi, 2012 |  |
| *Thysanolaena latifolia* (Roxb. ex Hornem.) Honda | 1 | 0.02 | Kantasrila, 2016 |  |
| **Polygalaceae** |  |  |  |  |
| *Polygala arillata* Buch.-Ham. ex D.Don | 2 | 0.04 | Kantasrila, 2016 |  |
| **Polygonaceae** |  |  |  |  |
| *Fallopia forbesii* (Hance) Yonek. & H.Ohashi | 1 | 0.02 | Srithi, 2012 |  |
| *Persicaria odorata* (Lour.) Soják | 3 | 0.05 | Field study  Gunsuwan, 2011 | 830, 962 |
| *Polygonum multiflorum* Gueldenst. | 1 | 0.02 | Srithi, 2012 |  |
| *Polygonum paleaceum* Wall. ex Hook.f. | 2 | 0.04 | Sukkho, 2008 |  |
| *Reynoutria japonica* Houtt. | 1 | 0.02 | Nuammee, 2012 |  |
| **Polypodiaceae** |  |  |  |  |
| *Nephrolepis falcata* (Cav.) C.Chr. | 2 | 0.04 | Anderson, 1993 |  |
| *Platycerium wallichii* Hook. | 1 | 0.02 | Kantasrila, 2016 |  |
| **Pontederiaceae** |  |  |  |  |
| *Pontederia vaginalis* Burm.f. | 1 | 0.02 | Anderson, 1993 |  |
| **Primulaceae** |  |  |  |  |
| *Ardisia polycephala* Wall. ex A.DC. | 2 | 0.04 | Sonnsupub, 2010 |  |
| *Embelia ribes* Burm.f. | 2 | 0.04 | Imchan, 2006;  Kantasrila, 2016 |  |
| *Embelia sessiliflora* Kurz | 7 | 0.13 | Field study  Sukkho, 2008;  Tangjitman, 2016;  Tovaranonte, 1998;  Trisonthi and trisonthi, 2009; | 1004 |
| *Embelia tsjeriam-cottam* (Roem. & Schult.) A.DC. | 1 | 0.02 | Sukkho, 2008 |  |
| *Maesa montana* A.DC. | 3 | 0.05 | Anderson, 1993;  Trisonthi and trisonthi, 2009 |  |
| **Pteridaceae** |  |  |  |  |
| *Adiantum philippense* L. | 1 | 0.02 | Nuammee, 2012 |  |
| *Pteris semipinnata* L. | 1 | 0.02 | Anderson, 1993 |  |
| **Ranunculaceae** |  |  |  |  |
| *Clematis smilacifolia* Wall. | 1 | 0.02 | Kaewdangsai, 2017 |  |
| *Thalictrum foliolosum* DC. | 3 | 0.05 | Srithi, 2012 |  |
| **Rhamnaceae** |  |  |  |  |
| *Ventilago denticulata* Willd. | 1 | 0.02 | Anderson, 1993 |  |
| *Ventilago harmandiana* Pierre | 1 | 0.02 | Prachuabaree, 2008 |  |
| *Ziziphus cambodiana* Pierre | 2 | 0.04 | Junsongduang, 2014;  Winijchaiyanan, 1995 |  |
| **Rosaceae** |  |  |  |  |
| *Agrimonia nepalensis* D.Don | 2 | 0.04 | Srithi, 2012 |  |
| *Agrimonia pilosa* Ledeb. | 1 | 0.02 | Field study | 798 |
| *Prunus cerasoides* Buch.-Ham. ex D.Don | 1 | 0.02 | Sukkho, 2008 |  |
| *Rubus alceifolius* Poir. | 2 | 0.04 | Nuammee, 2012;  Tangjitman, 2014 |  |
| *Rubus leucanthus* Hance | 1 | 0.02 | Field study | 977 |
| *Rubus sumatranus* Miq. | 1 | 0.02 | Field study | 988 |
| **Rubiaceae** |  |  |  |  |
| *Dimetia ampliflora* (Hance) Neupane & N.Wikstr. | 1 | 0.02 | Kantasrila, 2016 |  |
| *Dimetia capitellata* (Wall. ex G.Don) Neupane & N.Wikstr. | 1 | 0.02 | Tovaranonte, 1998 |  |
| *Hedyotis acutangula* Champ. ex Benth. | 5 | 0.09 | Field study | 943, 1024 |
| *Hedyotis pruinosa* Wight & Arn. | 1 | 0.02 | Sonnsupub, 2010 |  |
| *Ixora cibdela* Craib | 2 | 0.04 | Anderson, 1993 |  |
| *Ixora henryi* H.Lév. | 1 | 0.02 | Kaewdangsai, 2017 |  |
| *Morinda citrifolia* L. | 1 | 0.02 | Junkhonkaen, 2012 |  |
| *Mussaenda sanderiana* Ridl. | 3 | 0.05 | Kantasrila, 2016;  Tangjitman, 2014 |  |
| *Paederia foetida* L. | 1 | 0.02 | Srithi, 2012 |  |
| *Paederia pilifera* Hook.f. | 7 | 0.13 | Field study  Srithi, 2012; | 626, 864 |
| *Psychotria yunnanensis* Hutch. | 1 | 0.02 | Kaewdangsai, 2017 |  |
| *Punica granatum* L. | 2 | 0.04 | Anderson, 1993;  Sukkho, 2008 |  |
| *Rubia cordifolia* L. | 1 | 0.02 | Kantasrila, 2016 |  |
| *Spermacoce exilis* (L.O.Williams) C.D.Adams ex W.C.Burger & C.M.Taylor | 1 | 0.02 | Chaunchom, 2011 |  |
| **Rutaceae** |  |  |  |  |
| *Citrus × aurantiifolia* (Christm.) Swingle | 4 | 0.07 | Kantasrila, 2016;  Sonnsupub, 2010;  Winijchaiyanan, 1995 |  |
| *Citrus × reticulata* Blanco | 1 | 0.02 | Sonnsupub, 2010 |  |
| *Citrus cavaleriei* H.Lév. ex Cavalerie | 1 | 0.02 | Kaewdangsai, 2017 |  |
| *Citrus hystrix* DC. | 1 | 0.02 | Winijchaiyanan, 1995 |  |
| *Citrus maxima* (Burm.) Merr. | 4 | 0.07 | Field study  Winijchaiyanan, 1995 | - |
| *Clausena excavata* Burm.f. | 9 | 0.16 | Field study  Kantasrila, 2016;  Nuammee, 2012 | 636, 839 |
| *Clausena harmandiana* (Pierre) Guillaumin | 1 | 0.02 | Prachuabaree, 2008 |  |
| *Clausena lenis* Drake | 1 | 0.02 | Junsongduang, 2014 |  |
| *Clausena wallichii* Oliv. | 3 | 0.05 | Field study | 762 |
| *Harrisonia perforata* (Blanco) Merr. | 1 | 0.02 | Junkhonkaen, 2012 |  |
| *Melicope glomerata* (Craib) T.G.Hartley | 7 | 0.13 | Anderson, 1993;  Chaunchom, 2011;  Kantasrila, 2016  Srithi, 2012 |  |
| *Melicope pteleifolia* (Champ. ex Benth.) T.G.Hartley | 2 | 0.04 | Tangjitman, 2014 |  |
| *Micromelum integerrimum* (Roxb. ex DC.) Wight & Arn. ex M.Roem. | 1 | 0.02 | Kaewdangsai, 2017 |  |
| *Micromelum minutum* (G.Forst.) Wight & Arn. | 1 | 0.02 | Kantasrila, 2016 |  |
| *Psilopeganum sinense* Hemsl. | 2 | 0.04 | Srithi, 2012 |  |
| *Zanthoxylum acanthopodium* DC. | 1 | 0.02 | Trisonthi and trisonthi, 2009 |  |
| **Salicaceae** |  |  |  |  |
| *Flacourtia indica* (Burm.f.) Merr. | 4 | 0.07 | Pongamornkul, 2003;  Tangjitman, 2016;  Winijchaiyanan, 1995 |  |
| **Santalaceae** |  |  |  |  |
| *Henslowia collettii* Gamble | 1 | 0.02 | Kantasrila, 2016 |  |
| *Henslowia sessilis* Craib | 1 | 0.02 | Kaewdangsai, 2017 |  |
| *Scleropyrum pentandrum* (Dennst.) Mabb. | 3 | 0.05 | Kaewdangsai, 2017;  Kantasrila, 2016 |  |
| *Viscum articulatum* Burm.f. | 1 | 0.02 | Pongamornkul, 2003 |  |
| **Sapindaceae** |  |  |  |  |
| *Cardiospermum halicacabum* L. | 1 | 0.02 | Srithi, 2012 |  |
| *Sapindus rarak* DC. | 1 | 0.02 | Field study | 813 |
| *Schleichera oleosa* (Lour.) Oken | 1 | 0.02 | Winijchaiyanan, 1995 |  |
| **Sapotaceae** |  |  |  |  |
| *Xantolis cambodiana* (Pierre ex Dubard) P.Royen | 1 | 0.02 | Tangjitman, 2014 |  |
| **Saururaceae** |  |  |  |  |
| *Houttuynia cordata* Thunb. | 24 | 0.43 | Field study  Anderson, 1993;  Imchan, 2006;  Kaewdangsai, 2017;  Nuammee, 2012;  Srithi, 2012;  Tangjitman, 2014;  Tovaranonte, 1998 | 753 |
| **Schizaeaceae** |  |  |  |  |
| *Lygodium flexuosum* (L.) Sw. | 5 | 0.09 | Anderson, 1993;  Mahawongsanan, 2008;  Srithi, 2012 |  |
| *Lygodium microphyllum* (Cav.) R.Br. | 2 | 0.04 | Field study | 845 |
| **Scrophulariaceae** |  |  |  |  |
| *Buddleja asiatica* Lour. | 3 | 0.05 | Field study  Kantasrila, 2016 | 1005 |
| **Selaginellaceae** |  |  |  |  |
| *Selaginella repanda* (Desv.) Spring | 1 | 0.02 | Anderson, 1993 |  |
| **Simaroubaceae** |  |  |  |  |
| *Eurycoma longifolia* Jack | 3 | 0.05 | Junkhonkaen, 2012  Tangjitman, 2014; |  |
| *Picrasma javanica* Blume | 3 | 0.05 | Pongamornkul, 2003;  Kantasrila, 2016;  Tangjitman, 2016 |  |
| **Smilacaceae** |  |  |  |  |
| *Smilax glabra* Roxb. | 1 | 0.02 | Kantasrila, 2016 |  |
| *Smilax luzonensis* C.Presl | 1 | 0.02 | Kaewdangsai, 2017 |  |
| *Smilax ovalifolia* Roxb. ex D.Don | 3 | 0.05 | Anderson, 1993 |  |
| **Solanaceae** |  |  |  |  |
| *Capsicum frutescens* L. | 1 | 0.02 | Tichachart, 2004 |  |
| *Physalis angulata* L. | 1 | 0.02 | Sonnsupub, 2010 |  |
| *Solanum aculeatissimum* Jacq. | 1 | 0.02 | Junkhonkaen, 2012 |  |
| *Solanum americanum* Mill. | 2 | 0.04 | Field study  Prachuabaree, 2008 | 736, 878 |
| *Solanum erianthum* D.Don | 5 | 0.09 | Field study  Nuammee, 2012;  Sukkho, 2008  Tovaranonte, 1998; | 604 |
| *Solanum lasiocarpum* Dunal | 1 | 0.02 | Sukkho, 2008 |  |
| *Solanum nigrum* L. | 1 | 0.02 | Tangjitman, 2016 |  |
| *Solanum torvum* Sw. | 4 | 0.07 | Gunsuwan, 2011;  Noitana et al., 2013;  Tichachart, 2004;  Tovaranonte, 1998 |  |
| *Solanum violaceum* Ortega | 5 | 0.09 | Junkhonkaen, 2012;  Kantasrila, 2016;  Pongamornkul, 2003;  Tichachart, 2004 |  |
| **Staphyleaceae** |  |  |  |  |
| *Dalrympelea pomifera* Roxb. | 2 | 0.04 | Kantasrila, 2016 |  |
| *Dalrympelea sphaerocarpa* (Hassk.) Nor-Ezzaw. | 1 | 0.02 | Anderson, 1993 |  |
| **Tectariaceae** |  |  |  |  |
| *Pteridrys syrmatica* (Willd.) C.Chr. & Ching | 2 | 0.04 | Junkhonkaen, 2012 |  |
| **Theaceae** |  |  |  |  |
| *Camellia sinensis* (L.) Kuntze | 2 | 0.04 | Pongamornkul, 2003;  Sukkho, 2008 |  |
| *Schima wallichii* (DC.) Korth. | 12 | 0.21 | Anderson, 1993;  Kantasrila, 2016  Moonjai, 2017;  Sukkho, 2008  Tangjitman, 2014;  Winijchaiyanan, 1995 |  |
| **Thymelaeaceae** |  |  |  |  |
| *Aquilaria crassna* Pierre ex Lecomte | 1 | 0.02 | Junkhonkaen, 2012 |  |
| **Torricelliaceae** |  |  |  |  |
| *Torricellia angulata* Oliv. | 3 | 0.05 | Srithi, 2012 |  |
| **Urticaceae** |  |  |  |  |
| *Boehmeria nivea* (L.) Gaudich. | 2 | 0.04 | Srithi, 2012;  Trisonthi and trisonthi, 2009 |  |
| *Debregeasia longifolia* (Burm.f.) Wedd. | 1 | 0.02 | Anderson, 1993 |  |
| *Girardinia diversifolia* (Link) Friis | 1 | 0.02 | Anderson, 1993 |  |
| *Leucosyke puya* (Hook.) den Baaker & Mabb. | 1 | 0.02 | Anderson, 1993 |  |
| *Procris repens* (Lour.) B.J.Conn & Hadiah | 2 | 0.04 | Field study | 661 |
| **Verbenaceae** |  |  |  |  |
| *Sambucus canadensis* L. | 2 | 0.04 | Field study  Sukkho, 2008 | 994 |
| *Sambucus javanica* Reinw. ex Blume | 5 | 0.09 | Field study  Anderson, 1993;  Junsongduang, 2014;  Puling, 2001;  Sukkho, 2008 | 868 |
| *Verbena officinalis* L. | 9 | 0.16 | Field study  Anderson, 1993 | 794 |
| *Viburnum sambucinum* Reinw. ex Blume | 1 | 0.02 | Sukkho, 2008 |  |
| **Violaceae** |  |  |  |  |
| *Viola inconspicua* Blume | 2 | 0.04 | Srithi, 2012 |  |
| *Viola yunnanensis* W.Becker & H.Boissieu | 1 | 0.02 | Srithi, 2012 |  |
| **Vitaceae** |  |  |  |  |
| *Causonis japonica* (Thunb.) Raf. | 2 | 0.04 | Field study | 664, 985 |
| *Cayratia pedata* Gagnep. | 1 | 0.02 | Kantasrila, 2016 |  |
| *Cissus bicolor* Domin | 2 | 0.04 | Tangjitman, 2014 |  |
| *Cissus discolor* Blume | 3 | 0.05 | Field study | 662, 805 |
| *Cissus repens* Lam. | 1 | 0.02 | Anderson, 1993 |  |
| *Leea indica* (Burm.f.) Merr. | 10 | 0.18 | Field study  Junsongduang, 2014;  Kaewdangsai, 2017;  Nuammee, 2012;  Srisanga, 1993;  Winijchaiyanan, 1995 | 973 |
| *Tetrastigma obovatum* Gagnep. | 2 | 0.04 | Field study | 675 |
| **Xanthorrhoeaceae** |  |  |  |  |
| *Aloe vera* (L.) Burm.f. | 1 | 0.02 | Anderson, 1993 |  |
| **Zingiberaceae** |  |  |  |  |
| *Alpinia calcarata* (Andrews) Roscoe | 1 | 0.02 | Gunsuwan, 2011 |  |
| *Alpinia galanga* (L.) Willd. | 5 | 0.09 | Gunsuwan, 2011  Nuammee, 2012;  Tangjitman, 2016;  Winijchaiyanan, 1995 |  |
| *Alpinia malaccensis* (Burm.f.) Roscoe | 5 | 0.09 | Kaewdangsai, 2017;  Kantasrila, 2016;  Sukkho, 2008;  Tangjitman, 2016 |  |
| *Alpinia zerumbet* (Pers.) B.L.Burtt & R.M.Sm. | 1 | 0.02 | Kantasrila, 2016 |  |
| *Amomum dealbatum* Roxb. | 1 | 0.02 | Nuammee, 2012 |  |
| *Curcuma aeruginosa* Roxb. | 1 | 0.02 | Nuammee, 2012 |  |
| *Curcuma campanulata* (Kuntze)’ kornick. | 1 | 0.02 | Srithi, 2012 |  |
| *Curcuma involucrata* (King ex Baker)’ kornick. | 2 | 0.04 | Srithi, 2012 |  |
| *Curcuma longa* L. | 2 | 0.04 | Sukkho, 2008 |  |
| *Curcuma parviflora* Wall. | 2 | 0.04 | Field study | 782, 873 |
| *Etlingera elatior* (Jack) R.M.Sm. | 1 | 0.02 | Nuammee, 2012 |  |
| *Hedychium coronarium* J.Koenig | 1 | 0.02 | Anderson, 1993 |  |
| *Hedychium flavum* Roxb. | 1 | 0.02 | Srithi, 2012 |  |
| *Kaempferia galanga* L. | 3 | 0.05 | Junkhonkaen, 2012;  Noitana et al., 2013;  Srithi, 2012 |  |
| *Kaempferia parviflora* Wall. ex Baker | 4 | 0.07 | Field study  Gunsuwan, 2011;  Nuammee, 2012 | 644 |
| *Kaempferia rotunda* L. | 2 | 0.04 | Srithi, 2012 |  |
| *Zingiber montanum* (J.Koenig) Link ex A.Dietr. | 1 | 0.02 | Kantasrila, 2016 |  |
| *Zingiber officinale* Roscoe | 19 | 0.34 | Field study  Anderson, 1993;  Imchan, 2006;  Junkhonkaen, 2012;  Srithi, 2012;  Sukkho, 2008 | - |
| *Zingiber ottensii* Valeton | 8 | 0.14 | Field study  Kantasrila, 2016;  Nuammee, 2012;  Pipitkul, 2001;  Srithi, 2012;  Sukkho, 2008 | - |
| *Zingiber purpureum* Roscoe | 2 | 0.04 | Srithi, 2012 |  |

**List of data sources**

Anderson, E.F. (1993) Plants and people of the golden triangle ethnobotany of the hill tribes of northern Thailand. Southwest Portland: Timber Press, Inc.

Chaunchom, P. (2011) Ethnobotany of Hmong at Ban Tabboek, Tambon Wangban, Amphoe Lomkao, Changwat Phetchabun, Forest Resource Administration. [Master thesis]: Kasetsart University.

Gunsuwan, P. (2011) Processes of transferring local wisdom on management of medicinal herbs diversity for traditional healing of Hmong Community : A Case study of Khun Chang Khian Village, Chang Phueak Sub-district, Mueang Chiang Mai District. [Master thesis]: Chiang Mai University.

Imchan, T. (2006) Hmong's Wisdom in The Utilization And Conservation of Medicinal Plants in Bo Phak Subdistict Chattrakarn District Phitsanulok Province. [Master thesis]: Naresuan University.

Junkhonkaen, J. (2012) Ethnobotany of Ban Bowee, Amphoe Suan Phueng, Changwat Ratchaburi. [Master thesis]: Kasetsart University.

Junsongduang, A. (2014) Roles and importance of sacred Forest in biodiversity conservation in Mae Chaem District, Chiang Mai Province. [PhD thesis]: Chiang Mai University.

Kaewsangsai S. (2017) Ethnobotany of Karen in Khun Tuen Noi Village, Mae Tuen Sub-district, Omkoi District, Chiang Mai Province. [Master thesis]: Chiang Mai University.

Kamwong, K. (2010). Ethnobotany of Karens at Ban Mai Sawan and Ban Huay Pu Ling, Ban Luang Sub-District, Chom Thong District, Chiang Mai Province. [Master thesis]: Chiang Mai University.

Kantasrila, R. (2016) Ethnobotany fo Karen at Ban Wa Do Kro, Mae Song Sub-district, Tha Song Yang District, Tak Province. [Master thesis]: Chiang Mai University.

Klibai, A. (2013) Self-care with indigenous medicine of long-eared Karen ethnic group: Case study Ban Mae Sin, Ban Kang Pinjai, Ban Slok, Wang Chin district, Phrae province. [Master thesis] Surin Rajabhat University.

Krueasan, D. (2000) Management, conservation and utilization of plant species by Hmong of Pah Poo Chom Village, Mae Taeng District, Chiang Mai Province. [Master thesis]: Chiang Mai University.

Mahawongsanan, A. (2008) Change of herbal plants utilization of the Pgn K'nyau : A case study of Ban Huay Som Poy, Mae Tia Watershed, Chom Thong District, Chiang Mai Province. [Master thesis]: Chiang Mai University.

Moonjai, J. (2017) Ethnobotany of ethnic group in Mae La Noi District, Mae Hong Son Province. [Master Thesis]: Chiang Mai University.

Noitana, P., Saipara, S., Khoomput, K. (2013) Ethnobotany of the Hmong at Nanoi district, Nan province. Naresuan Phayao Journal 6:3, 7.

Nuammee, A. (2012) Ethnobotany of Hmong in Ban Pang Chang, Tambon Pong, Amphoe Santisuk, Changwat Nan. [Master thesis]: Chulalongkorn University.

Pipitkul, S. (2001) Medicinal plant utilization for the living of hill tribes at Doi Musoe, Tak Province. [Master thesis]: Mahidol University.

Prachuabaree, L. (2008) Medicinal plants of Karang hill tribe in Baan Pong-lueg, Kaeng Krachan District, Phetchaburi Province. [Master thesis]: Silpakorn University.

Pongamornkul, W. (2003) An ethnobotanical study of the Karen at Ban Yang Pu Toh and Ban Yang Thung Pong, Chiang Dao district, Chiang Mai province. [Bachelor degree dissertation]: Chiang Mai University.

Puling, W. (2001) Ethnobotany of Karen for studying medicinal plants at Angka Noi and Mae Klang villages, Chomthong district, Chiang Mai. [Bachelor degree dissertation]: Chiang Mai University.

Sonsupub, B. [2010] Ethnobotany of karen community in Raipa village, Huaykhayeng subdistrict, Thongphahpume district, Kanchanaburi province. [Master thesis]: Kasetsart University.

Srisanga P (1993) Ethnobotanical study of the Hmong Lai at Mae Sa Mai Village, Chiang Mai. Chiang Mai University, Chiang Mai.

Srithi, K. (2012) Comparative ethnobotany in Nan province, Thailand. [PhD thesis]: Chiang Mai University.

Sukkho, T. (2008) A survey of medicinal plants used by Karen people at Ban Chan and Chaem Luang Subdidtricts, Mae Chaem district, Chiang Mai province. [Master thesis]: Chiang Mai University.

Sutjaritjai, N., Wangpakapattanawong, P., Balslev, H. & Inta, A., Sutjaritjai, N., P. Wangpakapattanawong, H. Balslev and A. Inta (2019). Traditional Uses of Leguminosae among the Karen in Thailand. Plants 8:12. https://doi.org/10.3390/plants8120600.

Tangjitman, K. (2014) Vulnerability prediction of medicinal plants used by Karen people in Chiang Mai province to climatic change using species distribution model (SDM). [Ph.D. thesis]: Chiang Mai University.

Tangjitman, K. (2016) Ethnobotany of the Karen at Huay Nam and Nong Ta Dang Villages, Tanaosri Subdistrict, SuanphuangDistrict, Ratchaburi Province. [Scientific report]: Muban Chombueng Rajabhat University.

Tichachart, C. (2004) Ethnobotany of hmong hilltribe in Tambon Kheknoi, Amphur Khaokor, Changwat Phetchabun, Economic Botany. [Master thesis]: Kasetsart University.

Tovaranonte, J. (1998) Ethnobotanical study of the Tai Lue, Hmong and Yao in Some areas of Nan Province. [Master thesis]: Chiang Mai University.

Trisonthi, S., Trisonthi, P. (1995) Ethnobotany of Karen in Mae Hae Nua village, Mae Na Jorn subdistrict, Mae Chaem district, Chiang Mai. [Scientific report]: Chulalongkorn University.

Trisonthi, C.; Trisonthi, P. (2009) Ethnobotanical study in Thailand, a case study in Khun Yuam district Maehongson province. Thai J Bot 1: 1-23.

Trisonthi C, Trisonthi P, Sookchot T. (2002) Ethnobotanical study of hill tribes in the Royal Project Development areas for documentation of medicinal plants (direct translated from Thai). [Scientific report]: Chiang Mai University.

Winijchaiyanan, P. (1995) Ethnobotany of Karen in Chiang Mai. [Master thesis]. Chiang Mai University.

Yarnvudhi A, Sungkaew S, Hermhuk S, Sunthornhao P, Onprom S (2016) Plant diversity and utilization on ethnobotany of local people at Hmong Doi Pui Village in Doi Suthep-Pui National Park, Chiang Mai Province. Thai Journal of Forestry. 35:3, 136-146.
